# Sensing of cell-associated HTLV by plasmacytoid dendritic cells is regulated by dense β-galactoside glycosylation

**DOI:** 10.1371/journal.ppat.1007589

**Published:** 2019-02-28

**Authors:** Sonia Assil, Nicolas Futsch, Elodie Décembre, Sandrine Alais, Antoine Gessain, François-Loïc Cosset, Renaud Mahieux, Marlène Dreux, Hélène Dutartre

**Affiliations:** 1 CIRI–Centre International de Recherche en Infectiologie, Univ Lyon, Inserm, U1111, Université Claude Bernard Lyon 1, CNRS, UMR5308, ENS Lyon, Lyon, France; 2 Epidémiologie et Physiopathologie des Virus Oncogènes, Institut Pasteur, Paris France; University of Wisconsin, UNITED STATES

## Abstract

Human T Lymphotropic virus (HTLV) infection can persist in individuals resulting, at least in part, from viral escape of the innate immunity, including inhibition of type I interferon response in infected T-cells. Plasmacytoid dendritic cells (pDCs) are known to bypass viral escape by their robust type I interferon production. Here, we demonstrated that pDCs produce type I interferons upon physical cell contact with HTLV-infected cells, yet pDC activation inversely correlates with the ability of the HTLV-producing cells to transmit infection. We show that pDCs sense surface associated-HTLV present with glycan-rich structure referred to as biofilm-like structure, which thus represents a newly described viral structure triggering the antiviral response by pDCs. Consistently, heparan sulfate proteoglycans and especially the cell surface pattern of terminal β-galactoside glycosylation, modulate the transmission of the immunostimulatory RNA to pDCs. Altogether, our results uncover a function of virus-containing cell surface-associated glycosylated structures in the activation of innate immunity.

## Author summary

Human T Lymphotropic virus type (HTLV) establishes persistent infections, leading to adult T-cell Leukemia, a life-threatening cancer in chronically-infected individuals. Viral persistence likely results from a failure of immune responses to eradicate viral replication, a least in part, by viral escape from innate immunity, and notably via decreased production of type I interferons (IFN-I) by infected cells. Plasmacytoid dendritic cells (pDCs) are known as robust producers of IFN-I in response to virus stimulation, thus bypassing the viral mechanisms to evade pathogen-sensing pathways in infected cells. However, HTLV particles are not detected in biological fluids of infected individuals, raising the question of the pDC-activating signal. Here, we demonstrate that pDCs produce IFN-I upon physical contacts with HTLV-infected cells. We show that pDCs sense surface associated-HTLV present with glycan-rich structure, referred to as HTLV-biofilm-like structure. Importantly, the sensing of infected cells by pDCs is modulated by the glycosylation pattern at the surface of infected cells. This newly ascribed regulation of innate immunity activation by cell surface-associated glycans might contribute to the differential activation levels of antiviral response to infected cells when their glycosylation profile is modified, such as for chronically infected cells or tumor cells.

## Introduction

Human T-Lymphotropic Virus type 1 (HTLV-1) infects over an estimation of 5–10 million people. HTLV-1 is mainly present in Japan, central Africa, Caribbean and South America [1,2]. After a long period of clinical latency, HTLV-1 infection leads, in a fraction of infected individuals, either to Adult T-cell Leukemia/Lymphoma (ATL) [3] an uncontrolled CD4^+^ T–cell proliferation of very poor prognosis, or to an inflammatory disorder named HTLV-1 Associated Myelopathy / Tropical Spastic Paraparesis (HAM/TSP) [4]. In chronically infected individuals, HTLV-1 provirus is mainly found in CD4^+^ T-cells, yet infected dendritic cells (DCs) are also detected [5,6]. Their function is subsequently altered *in vivo* [6–8], thereby most likely contributing to viral pathogenesis.

Viral persistence leading to chronic infection and its associated diseases implies that innate and adaptive immune responses fail to eliminate HTLV-1 infected cells, possibly because HTLV-1 has evolved efficient strategies to escape immune pathways [9]. Type-I interferons (referred herein to as IFN-I, i.e., IFNα and β) are key mediators of innate immunity. They induce the expression of IFN-stimulated genes (ISGs) that suppress viral spread at different stages of the viral cycle, and stimulate the onset of adaptive immune responses. The IFN-I response is initiated *via* the recognition of pathogen-associated molecular patterns (PAMPs) by pattern-recognition receptors (PRRs), including the Toll like receptors (TLRs) [10]. Like virtually all viruses [11], HTLV-1 inhibits several steps of the PRR-induced pathways [12–14], and as a consequence, blunts IFN-I induction and signaling [15,16], leading to very limited production of IFN-I by infected cells.

Because the acute phase of the infection is asymptomatic, very little is known regarding host innate responses in HTLV-1-infected individuals. Nonetheless, indirect evidence infers that the IFN-I response exerts an antiviral action against HTLV-1. First, while not easily detectable *in vivo* [17], viral proteins expression is induced in T-lymphocytes isolated from infected patients when cultured *ex vivo* [18], likely as a result of the relief from *in vivo* repression. Consistently, culture of HTLV-1-infected cells with IFN-β-expressing stromal cells represses viral protein expressions [18]. Second, exogenous IFN-I decreases viral protein translation *in vitro*, and protects lymphocytes from *de novo* infection [19]. Thus, IFN-I-mediated antiviral control of HTLV-1 infection is likely to occur *in vivo*. Nonetheless, the cell type that produces IFN-I during infection remains enigmatic.

Plasmacytoid dendritic cells (pDCs) act as sentinels of viral infection, as they are the major IFN-I producers *in vivo* [20], being 1000-fold more potent for IFN-I production as compared to other cell types [20]. They predominantly recognize viral nucleic acids, *i*.*e*. single-stranded RNA and non-methylated CpG-containing DNA, by TLR7 and TLR9, respectively [21]. Cell-free HTLV-1 particles, when added at high concentration, were shown to induce IFN-I production by pDCs *in vitro*, in a TLR7-dependent manner [22]. Nonetheless, cell-free viruses are undetectable in the plasma of HTLV-1-infected individuals, which leaves open the question of the modality of pDC activation *in vivo*. Importantly, we and others recently revealed that cell contacts are required for efficient pDC activation by evolutionary divergent RNA viruses belonging to distinct families, such as *Flaviviridae*, *Picornaviridae*, *Arenaviridae*, *Retroviridae*, *and Togoviridae* [23–31]. Transfer of immunostimulatory viral RNAs from infected cells to pDCs was further shown to involve carriers in the form of non-infectious and/or non-canonical viral particles, including exosomes [25,27,29] and immature virus particles [24].

Interestingly, cell-cell transmission of viral material is reminiscent of HTLV cell-cell transmission [32], which is the only efficient way to infect new target cells. HTLV-1 viral transmission occurs through the transfer of neo-synthesized HTLV-1 virions via a virological synapse formed at the cell contact [33], and/or infectious viral particles embedded at the surface of infected cells within an extracellular matrix components (ECM)-rich structure [34]. The latter is referred to as the HTLV-1 biofilm-like structure [34]. This HTLV-1 biofilm-like structure has been further defined as the minimal infectious structure allowing viral transmission [35]. Importantly, the role of the cell surface associated virus within biofilm-like structure in the activation of the innate immune response is still unknown.

Here, we demonstrate that the pDC-mediated IFN-I response requires physical contacts with HTLV-infected cells. Moreover, we show that HTLV-1 biofilm-like structure represents the minimal virally induced-structure able to trigger an IFN-I response by pDC, and thus recapitulating pDC activation induced by contact with infected cells. Further, comparison of a panel of HTLV1/2 infected cells reveals that pDC-mediated IFN-I response inversely correlates with the ability of the HTLV-infected cells to transmit infectivity and with their surface glycosylation pattern. Indeed, we show that the density of terminal β-galactoside glycosylation at the surface of infected cells regulates IFN-I production by pDC. Altogether, our results highlight an unforeseen function of virus-containing cell surface-associated structures in the activation of pDCs by cell contacts, as well as its fine-tuning by the glycosylation pattern at the surface of the sensed infected cells.

## Results

### pDC IFN-I production induced by cell contact-dependent sensing of HTLV-1 infected cells

**Fig 1:**
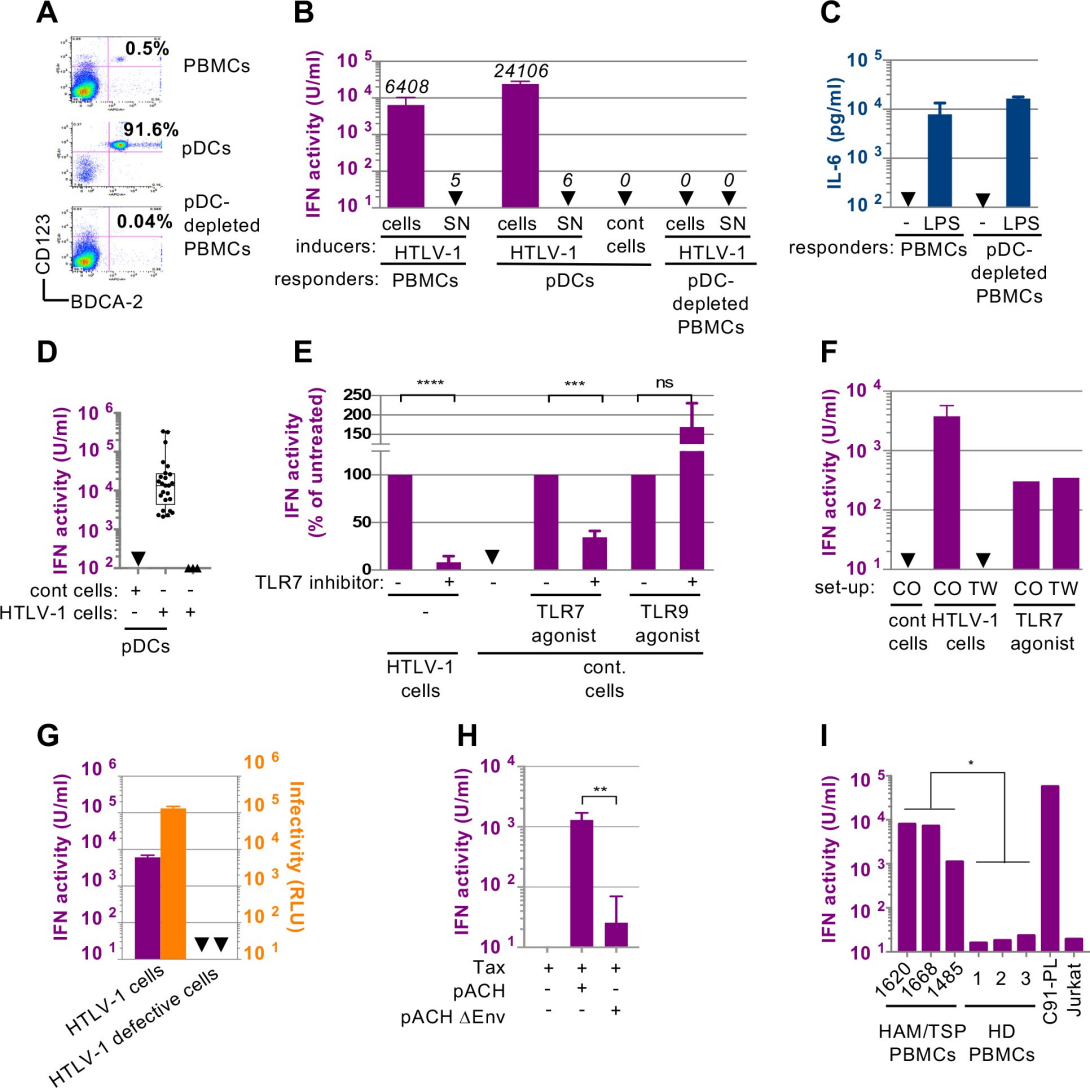
HTLV-1 infected cells robustly trigger IFN-I response by pDCs in a TLR7- and cell-cell contact dependent manner. **A.** Representative FACS analysis of pDC isolation and depletion from PBMCs using the CD123 and BDCA-2 pDC selective markers. **B.** Representative quantification of IFN-I activity in the supernatants of PBMCs, isolated pDCs and pDC-depleted PBMCs (responders) co-cultured with HTLV-1 infected cells (HTLV-1; C91-PL cells) or uninfected cells (cont cells, Jurkat cells), or their supernatants (SN) as indicated (inducers). The results are expressed as unit (U)/mL (one unit corresponding to 10–20 pg of recombinant IFN-α 2A). Results are representative of 3 independent experiments (means of experimental triplicates ± standard deviation; SD). Arrows indicate results below the threshold detection of the assay (*i*.*e*. 2.5 U/mL). **C.** Quantification of IL6 in the supernatants (SN) of PBMCs, pDC-depleted PBMCs treated or not with LPS, as indicated (responders). Arrows indicate results below the threshold detection of the assay (*i*.*e*., 4 pg/mL). **D.** Quantification of IFN-I activity (as in B) secreted by pDCs (2x10^4^) isolated from the blood of a cohort of healthy donors (n = 27) co-cultured with HTLV-1 infected cells (C91-PL, 2x10^4^) or uninfected (cont) cells (Jurkat, 2x10^4^), or secreted by HTLV-1 infected cells (C91-PL, n = 4) in absence of pDCs. Graph shows minimum, maximum and median values as well as q1-q3 quartiles. **E.** Quantification of IFN-I activity in the SNs of pDCs that were pre-incubated, or not, with TLR7 inhibitor (IRS661; 0.35 μM), as indicated, then co-cultured with infected cells (HTLV-1 cells; C91-PL) or with control cells (cont.; Jurkat cells) and stimulated by agonist of TLR7 (R848; 50 ng/mL) or of TLR9 (ODN2216; 0.1 μM). Results are expressed relative to IFN-I activity determined in the absence of TLR7 inhibitor, set at 100 (means ± SD; n = 3). Asterisks indicate statistically significant differences calculated using unpaired t-test: *** p< 0.001; **** p< 0.0001; ns: non significant. **F.** Quantification of IFN-I activity in the SNs of pDCs co-cultured with HTLV-1 infected cells either seeded together (CO) or separated by a 0.4 μm transwell membrane (TW). As controls, pDCs were treated with TLR7 agonist (as in E) in the same set-up. Results are representative of 4 independent experiments in triplicate (means ± SD). **G.** Paralleled quantification of IFN-I activity produced by pDCs (left axis) upon co-culture with HTLV-1-infected cells (C91-PL) versus C8166 HTLV-1-infected (labeled as defective cells) and the infectivity transmission levels to naïve Jurkat-LTR-Luc cells upon co-culture with the same cells (right axis). RLU, relative light unit (mean ± SD, 2 independent experiments). **H.** Quantification of IFN-I activity in the SNs of pDCs co-cultured with Jurkat cells transfected prior to coculture either with pACH WT molecular clone or with a clone lacking the envelope glycoprotein (pACH ΔEnv), along with Tax-expressing plasmid to increase viral expression (mean ± SD; 3 independent experiments). Asterisks indicate statistically significant differences calculated using unpaired t-test: ** p< 0.01. **I**. Quantification of IFN-I activity in the SNs of pDCs co-cultured either with PBMCs obtained from 3 independent HAM/TSP patients, or with PBMCs from 3 independent healthy donors. HTLV-1 infected cells (C91-PL) or uninfected cells (Jurkat) were used as controls. Results represent one experiment performed in triplicates. Asterisks indicate statistically significant differences calculated using unpaired t-test: * p< 0.05.

We first determined the production of type I IFN (referred to as IFN-I) by PBMCs and pDCs upon recognition of infected cells as compared to cell-free virions present in supernatant (SN) of infected cell lines (**Fig 1**). pDCs, representing 0.2–0.5% of total PBMCs, were isolated from healthy blood donors with >91% of purity (**Fig 1A,** middle panel), consistently with our previous reports [24,25,30]. PBMCs or purified pDCs (referred to as responders) were co-cultured with HTLV-1 chronically infected cells, *i*.*e*., C91-PL cell line [36], (referred to as inducer). These HTLV-1-infected cells induced a potent IFN-I response by both PBMCs and purified pDCs, when in physical contact (**Fig 1B**). In sharp contrast, cell-free viruses present in the supernatant from HTLV-1-infected cells (approximately 10–25 ng/mL of the HTLV-1 capsid p19^gag^, *i*.*e*., representing the viral concentration reached in the supernatant of inducer cells at the time of coculture) failed to induce very low, or undetectable levels of IFN-I production (**Fig 1B**, around 5 U/mL when detected, or below the detection limit).

Next, we tested the contribution of pDCs relative to other PBMC cell types in the IFN-I response to HTLV-1 infected cells. Depletion of pDCs from PBMCs (**Fig 1A**, lower panel) abrogated the response to HTLV-1 infected cells (**Fig 1B**). We controlled that pDC-depleted PBMCs and PBMCs produced comparable levels of IL-6 after LPS stimulation, confirming that pDC depletion did not impair PBMC responsiveness (**Fig 1C**). pDCs obtained from 27 donors, reproducibly demonstrated robust IFN-I responses to HTLV-1 infected cells (**Fig 1D;** median value of 13 400 U/mL), albeit with some donor-to-donor variations. Of note, HTLV-1 infected cells alone did not produce IFN-I (**Fig 1D**). Together, these results indicate that pDCs are the main, if not exclusive, IFN-I producers among PBMCs in response to the contact with HTLV-1-infected cells.

Next, we tested whether pDC sensing of HTLV-1 infected cells involves TLR7, a sensor of single-stranded RNA. Inhibition of TLR7 recognition using a competitive inhibitor significantly decreased the IFN-I response to HTLV-1-infected cells (**Fig 1E**). The specificity of TLR7 inhibitor was validated by the inhibition of IFN-I production triggered by a TLR7 agonist but not by a TLR9 agonist, as expected (**Fig 1E**). These results suggested that pDCs sense HTLV-1-infected cells via TLR7, implying that HTLV-1 viral RNA is likely the immunostimulatory signal. Since IFN-I production by pDCs following incubation with cell-free viruses was not or barely detectable, we hypothesized that cell contacts are required for pDC activation. We thus measured IFN-I production when pDCs were physically separated from HTLV-1-infected cells by a 0.4μm permeable membrane (**Fig 1F**, TW). The absence of physical contact between inducer and responder cells abrogated IFN-I production (**Fig 1F**). We controlled that pDC responsiveness was maintained in this experimental setting, as pDCs produced similar amounts of IFN-I upon TLR7 agonist stimulation when cultured in transwell chambers or not (**Fig 1F**). This demonstrated that pDC contact with infected cells is required to trigger IFN-I production.

Exosomes have been involved in the transfer of immunostimulatory RNAs to pDCs for other viruses [25,27,29,37] and HTLV-1 infected cells are known to produce exosomes [38]. To test whether exosomes are involved in the transfer of the HTLV-1 immunostimulatory signals, we used the C8166 HTLV-1 cell line, which is impaired for expression of the structural proteins Gag and Env and thus do not produce infectious viral particles [39], as confirmed by absence of infectivity transmission to Jurkat-LTR-Luc reporter cell line (**Fig 1G**). While C8166 HTLV-1 cells retain the capacity to produce the Tax regulatory protein, and exosomes that contain several viral mRNAs [38], they failed to induce IFN-I production by co-cultured pDCs (**Fig 1G**). This inferred that the transmission of activating signal to pDCs likely requires Env gp46 and/or Gag mediated extracellular export of viral RNA, rather than exosomal export of viral RNAs. To address the importance of Env gp46 in pDC IFN-I response, we tested pDC activation upon co-culture with Jurkat cells transfected with the WT HTLV-1 molecular clone (*i*.*e*., pACH) or with the counterpart molecular clone lacking the envelope glycoprotein (*i*.*e*., pACH-ΔEnv). As expected, Env gp46 was not expressed when Jurkat cells were transfected with the ΔEnv molecular clone, while p19^gag^ levels were similar (**Figs 1 and S1A**). Cells harboring WT but not ΔEnv molecular clone or only Tax expressing vector induced a robust IFN-I production by co-cultured pDCs (**Fig 1H**).

Next, we tested whether primary HTLV-1 infected cells from HAM/TSP patients were also able to induce IFN-I production by pDCs. As HTLV-1 infected cells isolated from the blood of patients do not express HTLV-1 [18], PBMCs from 3 HAM/TSP patients were first cultured in presence of IL-2 and PHA to induce viral re-expression. This was controlled by p19^gag^ detection (**S1B Fig**). Viral re-expression was observed in all patient samples, with some donor-to-donor variation as expected (**S1B Fig**). These cells were then co-cultured with pDCs. PBMCs from the 3 independent HAM/TSP patients significantly induced pDC IFN-I production (**Fig 1I**), as opposed to the absence of response to PBMCs from healthy donors used as controls.

We then aimed at determining whether pDCs are susceptible to HTLV-1 infection as previously reported [5], in our experimental setup leading to IFN-I production (*i*.*e*., within 24h-incubation with HTLV-1 infected cells). The productive infection of pDCs at the end of co-culture with HTLV-1 infected cells was assessed by the detection of Tax, as we previously reported [32]. In contrast to monocytes-derived dendritic cells (MDDCs), Tax expression by pDCs was not readily detected 24h after co-culture with HTLV-1 infected cells (**S2A Fig**). Thus, this suggests that pDC IFN-I response to HTLV-1 infected cells does not involved a productive infection. Altogether, our results demonstrated that pDCs sense HTLV-1 infected cells by Env gp46-mediated transmission of pDC-activating signal by cell contact leading to robust IFN-I response via TLR7-induced signaling.

### Role of HTLV receptors in the sensing of HTLV-infected cells by pDCs

**Fig 2:**
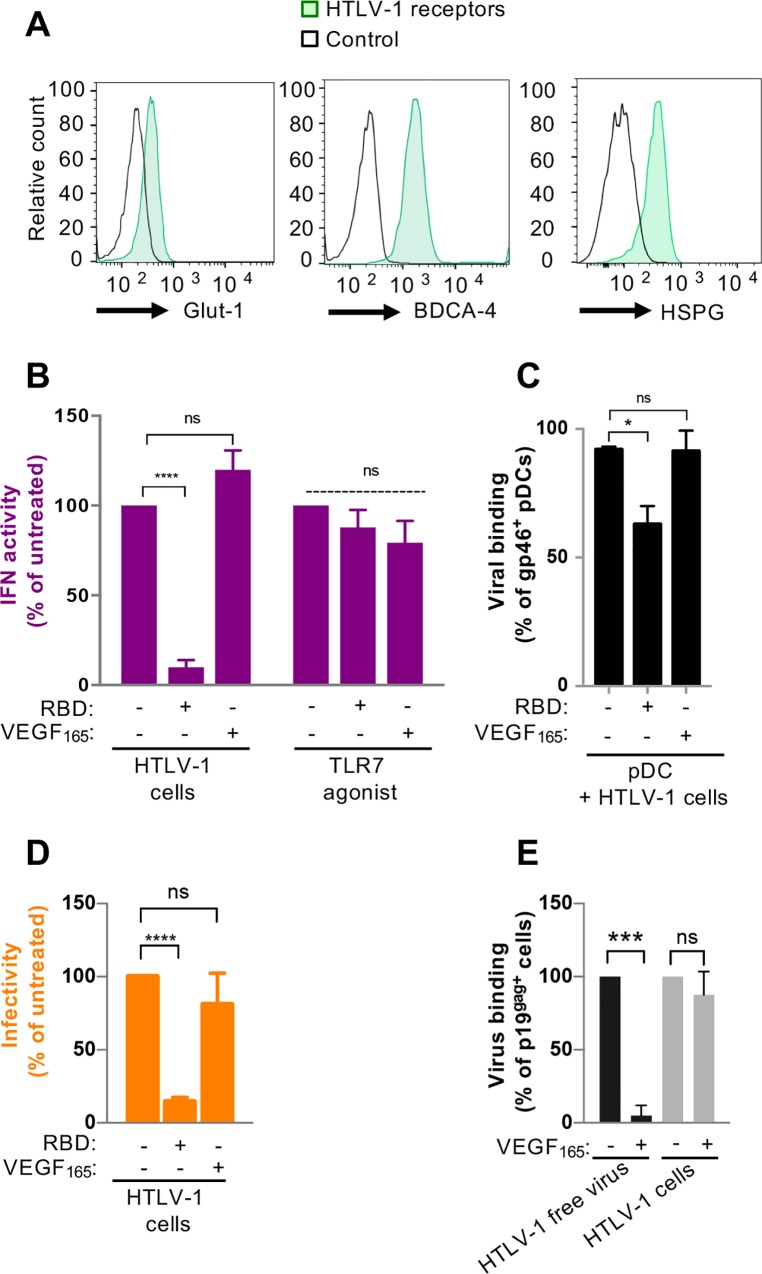
The HTLV-1 receptor Glut-1 is involved in pDC IFN-I production triggered by the sensing of HTLV-1 infected cells, but not Neuropilin-1/BDCA-4. **A.** Assessment by FACS of the surface expression of the HTLV-1 receptors Glut-1 (revealed with Glut-1.RBD.GFP and controlled with unstained cells), NRP-1/BDCA-4 (revealed with mAb and controlled with IgG isotype) and HSPG (revealed with mAb and controlled with IgG isotype). (representative of 3 independent experiments). **B-D.** Impact of Glut-1 binding competitor (RBD, 5 μl/10^5^ cells) or NRP-1/BDCA-4 binding competitor (VEGF_165_, 100 ng/mL) on IFN-I activity in SNs of pDCs co-cultured with HTLV-1-infected cells (C91-PL) (mean ± SD; 5 independent experiments) (B), viral binding was determined by flow cytometry after Env gp46 detection on pDCs surface (mean ± SD, 3 independent experiments) (C), and infectivity transmission levels (mean ± SD; 3 independent experiments) (D), determined as in Fig 1. The results in (C) and (D) are expressed as percentages relative to untreated co-cultures. Asterisks indicate statistically significant differences calculated using ANOVA followed by Sidak’s multiple comparison test: * p> 0.05, **** p< 0.0001; ns: non significant. **E.** Viral binding as determined by flow cytometry after p19^gag^ detection on Jurkat target cells upon exposure to HTLV-1 cell-free viruses or upon co-culture with HTLV-1 infected cells in the presence or not of NRP-1/BDCA-4 binding competitor (VEGF_165_, 80 ng/mL). Jurkat cells were differentiated from HTLV-1 infected cells based on their size (see **S2C Fig**). The results are expressed as percentage relative to untreated conditions (mean ± SD; 2–3 independent experiments). Asterisks indicate statistically significant differences calculated using ANOVA followed by Sidak’s multiple comparison test: *** p<0.001; ns = non significant.

The capture of HTLV-1 cell-free virus by target cells involved binding of Env gp46 to NRP-1/BDCA-4 in cooperation with HSPG [40] and then to Glut-1 [41]. The latter also serves as the receptor mediating fusion of HTLV envelope with the cellular membrane [42]. NRP-1/BDCA-4, Glut-1 and HSPG are all readily expressed at the pDC surface (**Fig 2A**). We thus sought to determine the contribution of these receptors in the transfer of the activating signal from the infected cells to the pDCs. Previous reports showed that the binding of HTLV-1 Env gp46 to its receptors is mediated by the receptor binding domain (RBD; the first 215 amino acids of gp46), and can thus be out-competed by recombinant RBD [41]. Competition with recombinant RBD significantly reduced IFN-I production by pDCs (**Fig 2B**), viral binding to pDCs (**Fig 2C** and **S2B Fig**) and viral transmission to reporter cells (**Fig 2D**). This suggests that pDC sensing requires HTLV-1 Env binding to its receptor(s). RBD comprises residues that have been specifically involved in NRP-1/BDCA4 (*i*.*e*., at the position 90-to-94) [40] and the 94-to-101 stretch known to be pivotal for Glut-1 binding and subsequent viral fusion [43]. Thus, it does not allow to discriminate between binding to NRP-1/BDCA-4 versus Glut-1. Nonetheless, binding of Env gp46 to NRP-1/BDCA4 can be prevented by addition of recombinant VEGF_165_, a known ligand of NRP-1/BDCA4, that interacts directly through a peptide stretch similar to the 90–94 sequence found in Env gp46 but also using an HSPG dependent manner [40]. Thus VEGF_165_ does not allow discriminating binding to NRP-1 versus HSPG. Competition with recombinant VEGF_165_ did not prevent the IFN-I production by pDCs (**Fig 2B**), viral binding to pDCs (**Fig 2C**) nor cell-cell viral transmission to reporter cells (**Fig 2D**), suggesting that NRP-1/BDCA-4 HSPG-mediated and/or direct binding may not be involved in HTLV-1 transfer by cell-cell contact. The effectiveness of VEGF_165_ competition was confirmed by the expected reduction of the binding of cell-free HTLV-1 virion to reporter cells measured by flow cytometry detection of p19^gag^ (**Fig 2E**), consistent with a previous report [40], whereas no competition by VEGF_165_ was observed in co-culture experiments with HTLV-1 infected cells (C91-PL) (**Fig 2E and S2C Fig**). VEGF_165_ and RBD treatment did not impair the pDC IFN-I response upon stimulation with a TLR7 agonist, thus ruling out non-specific effects of recombinant RBD and VEGF_165_ on pDC responsiveness (**Fig 2B**). Altogether, these results suggested that both transmission of infection to target cells after cell-cell contact as well as HTLV-1 sensing by pDCs require Env gp46 interaction with at least Glut-1.

### pDCs respond to HTLV-1 infected cells via sensing of the viral biofilm-like structure

**Fig 3:**
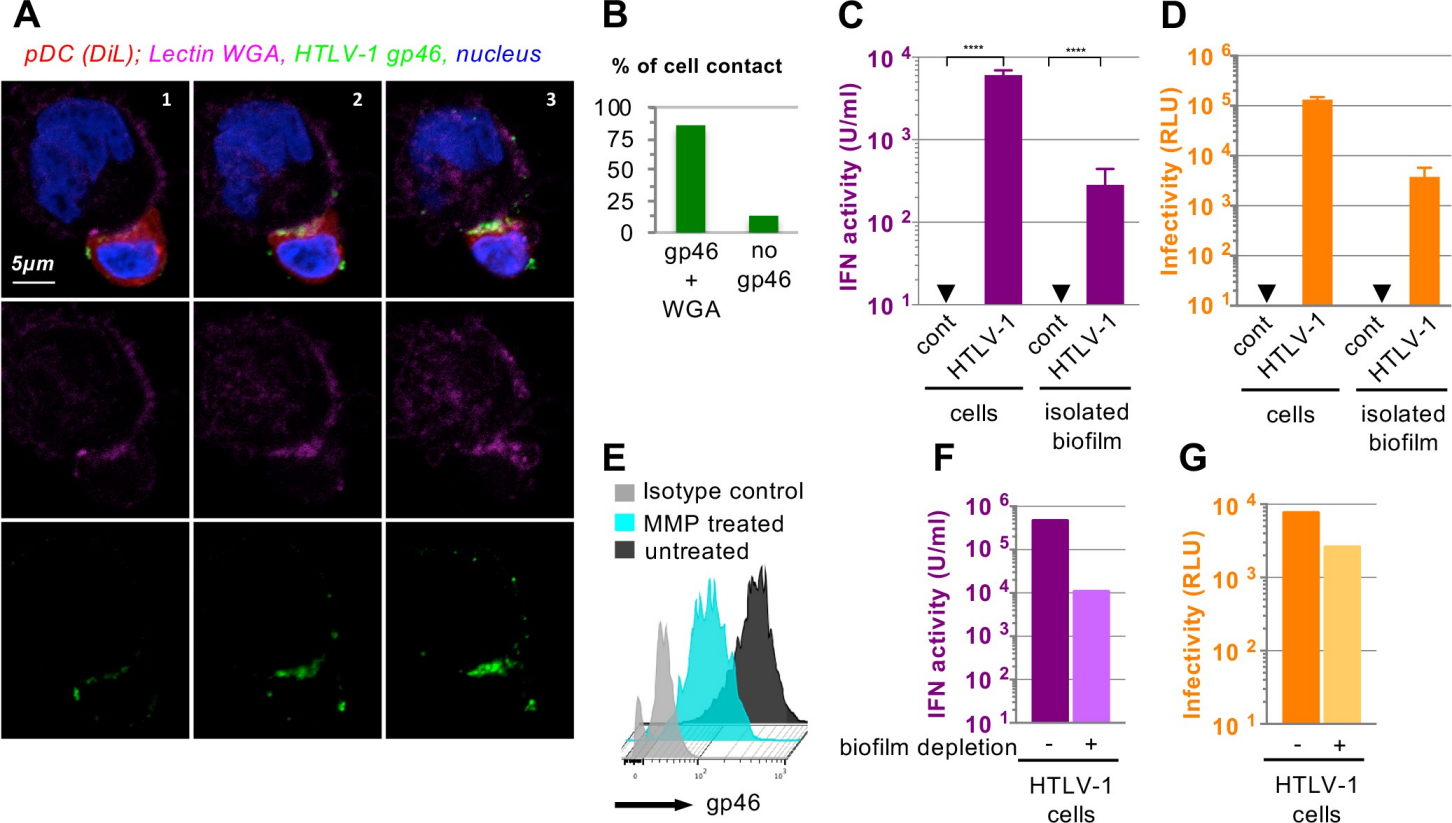
HTLV biofilm-like structures trigger a robust activation of pDC-IFN-I response. **A.** Representative confocal imaging of pDC/HTLV-1 infected cell contact with 3 consecutive Z-stack sections (left to right, all immunodetections were performed without permeabilization). Upper panels, confocal analysis of HTLV-1 Env gp46 (green) that form cluster at the contact between HTLV-1 infected cells and pDCs (stained with DiI, a lipophilic dye, red). Carbohydrate-rich structures are stained by the WGA lectin (Triticum vulgaris, purple), and nuclei are stained with Hoescht (blue). Middle and lower panels, single detections of WGA lectin and HTLV-1 Env gp46, respectively. **B.** Quantification of the presence or not of Env gp46 and WGA co-clusters at the cell-cell contact (analysis of 16 cell-cell contacts in 3 independent experiments). **C-D.** Parallel quantification of IFN-I activity in SNs of pDCs co-cultured with HTLV-1-infected cells (C91-PL) versus biofilm-like structures isolated from HTLV-1-infected cells (C91-PL) and infectivity transmission levels, determined as in Figs 1G and 2D (mean ± SD; 3 independent experiments performed with similar viral capsid levels in the isolated HTLV-1 biofilm-like structure as determined by p19^gag^ levels, *i*.*e*. 2.9 ng). Asterisks indicate statistically significant differences calculated using ANOVA followed by Sidak’s multiple comparison test: **** p<0.0001. **E.** Surface Env gp46 detection on HTLV-1 infected cells (C91-PL) treated or not with metalloproteinase 9 (MMP-9; 20 nM). **F-G**. Parallel quantifications of IFN-I activity in SNs of pDCs co-cultured with HTLV-1-infected cells (C91-PL) treated or not with MMP-9 as in C, and infectivity transmission levels to Jurkat reporter cells co-cultured with HTLV-1-infected cells (C91-PL) treated or not with MMP-9 determined as in Fig 2D. The results are representative of 3 independent experiments.

Previous reports showed that HTLV-1 virions are present at the cell surface embedded within carbohydrate-rich elements, referred to as a viral biofilm-like structure [34], and involved in the infectivity transmission [34,35]. Since pDCs respond to HTLV-1-infected cells upon physical contact, we first determined whether HTLV-1 biofilm-like structure was present at the contact site between pDCs and HTLV-1-infected cells. Confocal microscopy analyses of pDC in contact with C91-PL cells revealed that HTLV-1 Env gp46 accumulates at the pDC/infected cell interface, together with carbohydrate-rich elements, known to be present in the viral biofilm-like structure [34], as revealed here by WGA lectin staining (**Fig 3A**). Of note, Env gp46 and WGA clusters were co-localized at the contact site for most of the analyzed pDC/infected cell contacts (**Fig 3B,** approx. 85%), suggesting that cell contacts are preferentially oriented toward these specific biofilm-like structures, or inversely that the biofilm-like structures are preferentially positioned at the contact site.

Next, we determined whether HTLV-1 biofilm-like structures could trigger pDC response. pDCs were cultured in the presence of HTLV-1 biofilm-like structures, isolated from infected cells as previously described [35]. Virus concentration in isolated biofilm-like structures ranged from 23.5 to 31.6 ng/mL of p19^gag^, a concentration similar to that found in the supernatant of infected cells. While cell-free virus-containing supernatants failed to induce IFN-I production by pDCs (**Fig 1B**), isolated biofilm-like structures significantly activated pDC IFN-I response (**Fig 3C**). This was specific to HTLV-1 infected cells, since similar isolation procedure from uninfected cells failed to activate the pDCs (**Fig 3C**, cont.), ruling out a putative non-specific activation by the experimental process (*e*.*g*., cellular debris). As expected, isolated biofilm-like structures from HTLV-1-infected cells transmitted infectious virions to Jurkat target cells, (**Fig 3D**). To further confirm that pDCs sense HTLV-1 biofilm-like structures, HTLV-1 biofilm-like structure was depleted using metalloprotease that digest the extracellular matrix [44] as previously described [34]. The metalloprotease treatment of HTLV-1-infected cells decreased the levels of surface envelope gp46 compared to untreated cells (**Fig 3E**), in association with a reduction in both IFN-I production by co-cultured pDCs and viral transmission to Jurkat-LTR-Luc reporter target cells (**Fig 3F and 3G**, and **S3A and S3B Fig**). This is shown for a representative experiment (*i*.*e*., pDCs from one blood donor co-cultured with biofilm-depleted HTLV-1 infected cells in **Fig 3F and 3G**) and for the means of independent experiments using pDCs from 3 blood donors (**S3A and S3B Fig**). Altogether, these results show that the HTLV-1 biofilm-like structure contains the immunostimulatory signal that triggers IFN-I production by pDCs.

### Enhanced sensing of HTLV-infected cells by pDCs in conjunction with increased pDC-infected cell contacts

**Fig 4:**
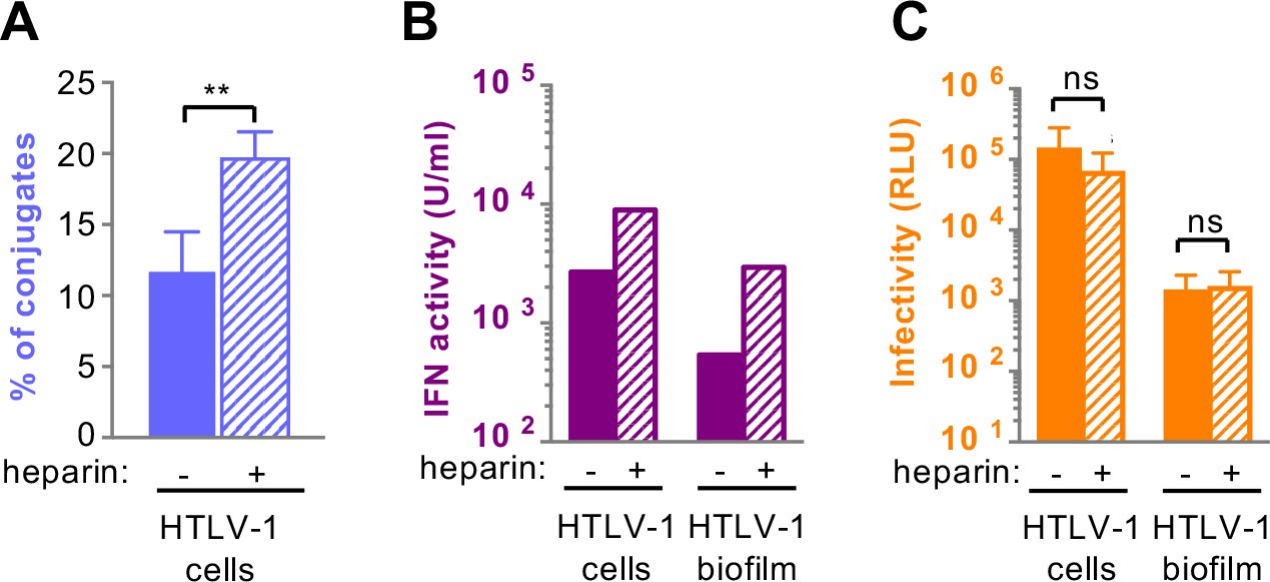
Heparin, increases pDC/infected cell contacts and pDC activation by both HTLV-infected cells and isolated HTLV biofilm-like structures. **A.** Quantification of the conjugates between CD123-stained pDCs and GFP-expressing HTLV-1 infected cells analyzed by imaging flow cytometry in co-cultures treated or not with heparin (50 U/ml). Results are expressed as percentages of pDC/HTLV-1 cell conjugates *i*.*e*., conjugates of, at least, one cell solely CD123^+^ and one cell GFP^+^, relative to the total number of single cells (GFP^+^ or CD123^+^) and the conjugates (mean ± SD; 3 independent experiments; n = 8000 events per condition). Asterisks indicate statistically significant differences calculated using ANOVA followed by Sidak’s multiple comparison test: ** p<0.01. **B.** Quantification of the effect of heparin treatment (50 U/mL) on IFN-I activity in SNs of pDCs co-cultured with HTLV-1-infected cells or HTLV-1-purified biofilm-like structures *i*.*e*., 2.9 ng as determined by p19^gag^ ELISA (mean of triplicates results; representative of 3 independent experiments). **C.** Quantification of infectivity transmission levels to naïve Jurkat-LTR-Luc cells in presence or not of heparin upon co-culture with the HTLV-1-infected cells or isolated HTLV-1 biofilm-like structures (mean ± SD; 3 independent experiments with similar viral capsid levels in the isolated HTLV-1 biofilm-like structure as determined by p19^gag^ levels). NS indicates statistically non-significant differences calculated using ANOVA followed by Sidak’s multiple comparison test.

Since HTLV-1 embedded in biofilm-like structure but not cell-free virons induced pDC IFN-I response, we next sought to examine the elements present in the biofilm-like structure that contribute to viral transmission and subsequent pDC activation. HTLV-1 biofilm-like structures contain ECM components and linkers, including collagen, agrin and heparan sulfate proteoglycans (HSPGs) [34]. As HSPGs are involved in cell-cell and cell-ECM interactions [45], we hypothesized that HSPGs present in HTLV-1 biofilm-like structure and/or in association with HSPGs at the pDC surface (**Fig 2A**, right panel) could favor cell-cell adhesion. To test this hypothesis, we used heparin, a polyanionic glycosaminoglycan that mimics the sulfate groups of HSPGs and that could thus act as a bridge to increase the pDC/infected cell contacts *via* the HTLV-1 biofilm-like structure. The impact of heparin on the frequency of cell conjugates formed between HTLV-1 infected cells and pDCs was analyzed by imaging flow cytometry (Image Stream X technology) (**S4A Fig**), as we previously established [24]. This quantitative analysis revealed that heparin significantly increased the frequency of pDC-HTLV-1-infected cell conjugates (**Fig 4A**). Consistently, heparin augmented pDC IFN-I production induced by HTLV-1 infected cells (**Fig 4B**). Importantly, heparin increased as well pDC activation induced by isolated HTLV-1 biofilm-like structures (**Fig 4B**). Similar results were obtained using blood samples from different donors (**S4B Fig**). HSPGs are known to act as attachment factors *via* an interaction with Env gp46 [46], thus heparin could compete for HTLV-1 binding and subsequent HTLV-1 infection. Nonetheless, similar heparin addition had no impact on the viral transmission to Jurkat reporter cells using either isolated HTLV-1 biofilm-like structures or HTLV-1-infected cells (**Fig 4C**). This contrasts with a previously reported impact of heparin in the context of distinct experimental procedure that showed that heparin, when added during biofilm isolation, reduced the ability of the isolated biofilm to infect Jurkat reporter cells [34]. The presence of heparin during biofilm isolation might have loosened the biofilm structure allowing a better exposure of the viral envelop to heparin competition. The lack of heparin competition both on infected cells and isolated biofilm (**Fig 4C**) demonstrated that under our experimental conditions, heparin did not compete with HSPGs for HTLV capture when HTLV-1 is embedded in an intact viral biofilm. Altogether, these experiments suggested that heparin increases pDC/infected cell contact as well as pDC transfer of the immunostimulatory signal from the isolated HTLV-1 biofilm-like structures and, likely as a consequence the potentiation of pDC IFN-I response, albeit additional effect can contribute as well. Importantly, the absence of modulation by these heparin treatments of infectivity transmission to target cells highlighted that the transfer of the immunostimulatory signal to pDCs features distinct regulatory mechanism(s) as compared to the infectivity transmission to other cell types.

### Sensing of HTLV-infected cells inversely correlates with viral transmission

**Fig 5:**
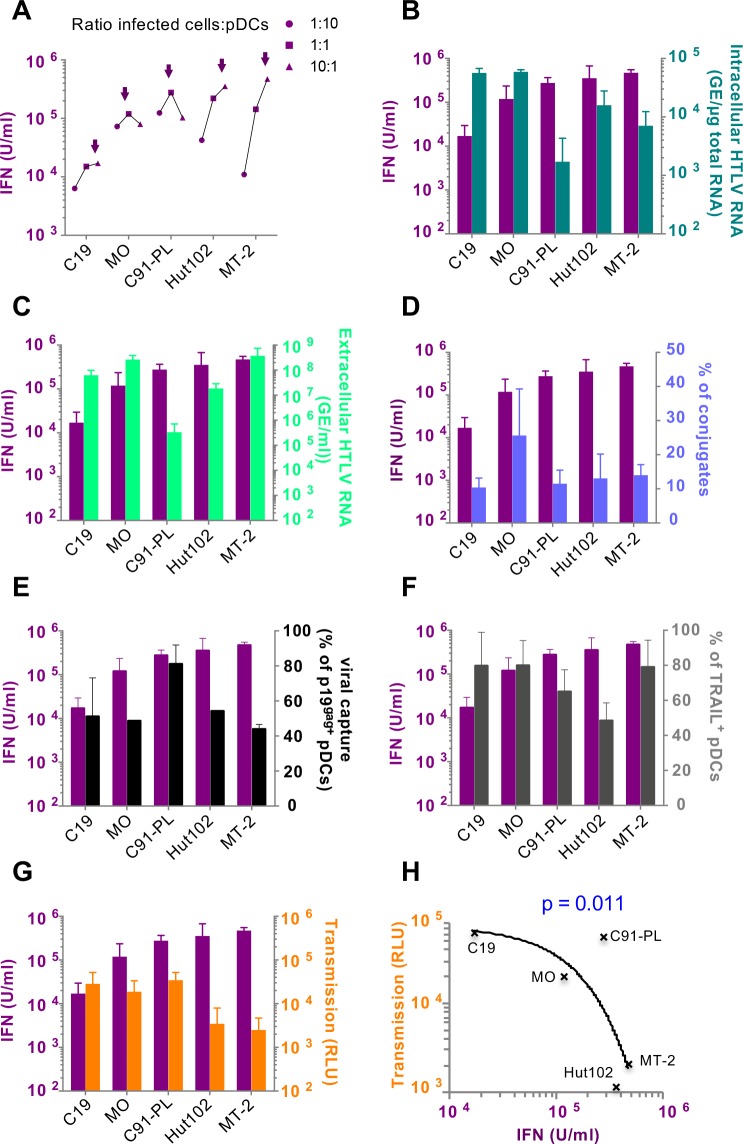
Levels of pDC IFN-I production triggered by HTLV infected cells inversely correlate to the efficiency of infectious viral transmission via cell-cell contact. **A.** IFN-I activity levels were quantified after co-culture of increasing number (2x10^3^; 2x10^4^; 2x10^5^) of HTLV-1 (C91-PL; Hut102; MT-2) or HTLV-2 infected cells (C19; MO) with pDCs (2x10^4^). The infected cells:pDC ratio is indicated on the right of the graph. Arrows indicate the maximum level of IFN-I activity for each cell line setting (mean of 3 independent experiments). **B-G.** Parallel representation of the maximum levels of IFN-I activity induced after co-culture of pDCs with HTLV-infected cells and of (**B**) intracellular viral RNA present in the cytoplasm of HTLV-infected cell lines (mean ± SD; 3 independent experiments); (**C**) viral RNA released in the supernatant of HTLV-infected cell lines after 24h of culture (mean ± SD; 3 independent experiments); (**D**) the percentage of cell contacts established between pDCs and HTLV-infected cells (mean ± SD; 2–4 independent experiments); (**E**) percentage of viral capture after 4h of co-culture with HTLV-1/2 infected cells as determined by p19^gag^ detection in pDCs (mean ± SD; 3 independent experiments); (**F**) percentage of TRAIL expression after 24h of co-culture detected on the pDCs surface (mean ± SD; 3 independent experiments) or (**G)** maximum infectivity levels as determined in **S5D Fig** (n = 3). **H.** Correlation curve of pDC-dependent IFN-I production and viral transmission and calculated p value.

Next, we sought to define the viral determinants and other cellular component(s) modulating the level of pDC response to infected cells, including the amounts of viral RNAs, cell contact efficiency and ability to transmit viral infectivity. To address these questions, we compared three HTLV-1 chronically infected cell lines (C91-PL, MT-2 and Hut102), known to produce different amounts of viral proteins [47] and two HTLV-2-infected cell lines (MO and C19). All HTLV-cell lines triggered IFN-I production by co-cultured pDCs, albeit at different levels ranking from lower to higher inducer cell lines, at the optimal pDC/infected cells ratios (**S5D Fig**), as follows: C19, MO, C91, Hut102 and MT-2 cell lines (**Fig 5A**). Neither differences in the amount of intracellular genomic RNA nor viral RNA released in the supernatant of infected cells was correlated to the observed differences in the induction of pDC IFN-I response (**Fig 5B and 5C and S5A and S5B Fig**). This suggests that the amount of RNA produced by the infected cells is not rate limiting for activation of pDCs.

Thus, we next assessed whether frequency of pDCs engaged in contacts with the different HTLV-infected cell lines regulated the intensity of pDC activation. The frequency of pDC conjugates with the different HTLV-infected cell lines was similar, except higher level for the HTLV-infected MO cell line (**Fig 5D**). Nonetheless, this higher frequency of cell conjugates with the HTLV-infected MO cell line did not translate into higher levels of IFN-I production (**Figs 5D and S5C**), suggesting that additional factor(s), other than pDC ability to establish contact with HTLV-infected cells, govern(s) the levels of pDC IFN-I response to HTLV-infected cells.

We thus tested whether variations of pDC induction by HTLV-infected cell lines might be explained by distinct mechanisms for viral capture by pDCs. To address this, we first evaluated viral binding and internalization in the pDCs upon co-culture with the different HTLV-1/2 cells lines by detection of intracellular p19^gag^ in the CD123^+^ pDCs population by FACS (**Fig 5E**). Except pDCs co-cultured with C91-PL, we observed no differences in virus binding on pDCs. This suggests that the reduced IFN response induced by C19 cells does not result from diminished HTLV-2 capture by pDCs. Furthermore, consistent with results obtained using cell-free virus [22], HTLV-1 infected cells induced TRAIL expression by co-cultured pDCs (**Fig 5F**), as did HTLV-2 infected cells (**Fig 5F**). This suggests that the reduced pDC IFN response to C19 cells is not associated with other impairment link in their ability to respond to virus. Furthermore, using C19 cells that induced the lowest pDC IFN-I production (**Fig 5A**), we showed that both the pDC response and viral transmission were significantly out-competed by recombinant RBD, but not by recombinant VEGF_165_ (**S6A–S6D Fig**), suggesting that different HTLV viral receptor usages are not likely responsible for the difference in pDC IFN-I response to the various cell lines. Additionally, pDC sensing of C19 cells was specifically inhibited by TLR7 inhibitor (**S6E Fig**). This rules out the involvement of other PRR that would induced lower IFN-I induction upon HTLV-2 sensing, as suggested for other viruses [28].

As we showed that pDCs are activated by cell-cell contacts with infected cells and *via* viral biofilm-like structures, we then compared the viral accumulation at the surface of the panel of HTLV-infected cell lines (**S6F Fig**). The p19^gag^ proteins were detected as patch/cluster at the surface of all infected cells, suggesting that the pDC activation is not directly linked to an absence of virus accumulation at the surface of the different HTLV-infected cell lines.

We next asked whether the level of pDC activation by the HTLV-infected cell lines correlate with their ability to transfer infectious virions to target cells. Regressive exponential correlation analysis revealed that IFN-I production by pDCs was inversely correlated with the ability of infected cells to transfer infectious virions (**Fig 5G and 5H,** p-value = 0,011). Altogether these results indicate that the sensing of HTLV-infected cells by pDCs is not strain-specific, and, importantly, inversely correlated to infectivity transmission to alternative target cells. It thus implies that pDC activation is likely modulated by other features of the infected cells.

### Sensing of HTLV-infected cells by pDCs inversely correlates with the density of β-galactoside glycosylation at the HTLV-infected cell surfaces.

**Fig 6:**
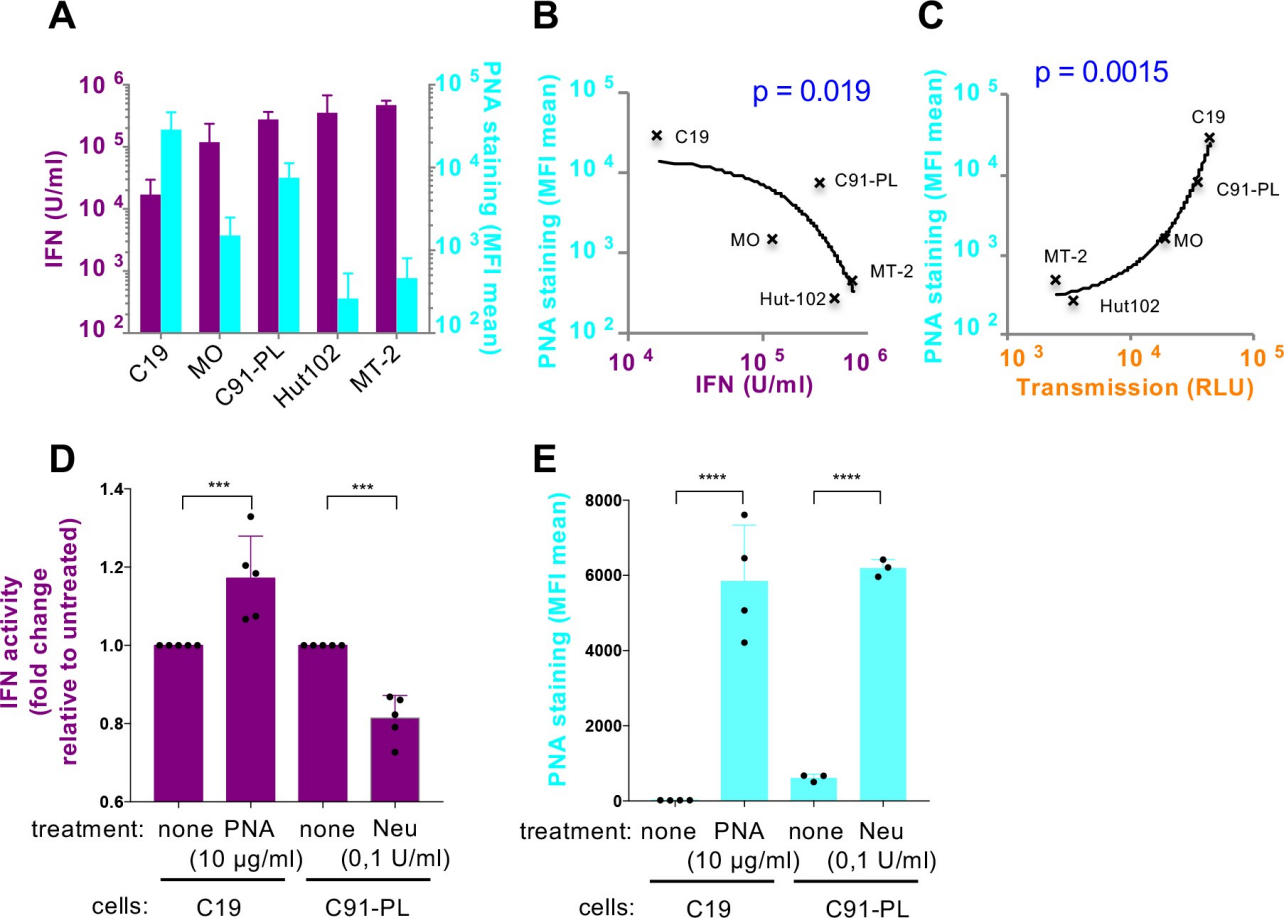
The differential surface glycan composition of HTLV-infected cells regulates the levels of pDC IFN-I production A. Parallel representation of the maximum levels of IFN-I production induced after co-culture of pDCs with HTLV-1-infected cells and the amount of surface PNA binding on HTLV-2 (C19 and MO) infected cell lines and HTLV-1 (C91-PL; Hut102 and MT-2) infected cell lines (mean ± SD; 5 independent experiments). **B-C.** Regression correlation curve of the amount of PNA binding at the surface of HTLV-infected cells and the maximum IFN-I production induced by the HTLV-infected cells (B) or the level of infectious viral transmission to naïve reported Jurkat LTR-Luc cells (C). Computes correlation p-values calculated with Spearman test are indicated. **D.** Quantification of IFN-I production by pDCs co-cultured with C19 or C91-PL infected cells treated or not with PNA (10 μg/ml) for 30 minutes or with neuraminidase (Neu, 0.1 U/ml) for 1h prior the co-culture, as indicated. Results are presented as fold changes relative to untreated cells (mean ± SD, 5 independent experiments). Asterisks indicate statistically significant differences calculated using unpaired t-test between treated versus untreated setting: *** p<0.001. **E.** FACS analysis of C19 stained or not with PNA (10μg/ml) and C91-PL infected cells treated or not with neuraminidase (Neu, 0.1 U/ml) for 1h before staining with PNA (mean ± SD; 4 and 3 independent experiments respectively). Asterisks indicate statistically significant differences calculated using paired t-test between treated versus untreated setting: **** p<0.0001.

Our results using heparin suggested that glycosylated proteins, including HSPGs are involved in the tethering of HTLV-1 stimulating signals to the pDC surface and/or its transfer, resulting in increased pDC IFN-I production. The density of surface glycosylation, including HSPGs [48], is known to be cell type specific [49]. We thus quantified cell surface glycosylation using various lectins known to bind different terminal glycosylation patterns (**S7A Fig**). The staining by Peanut agglutinin lectin (PNA), which bind to oligosaccharide structures with terminal β-galactose residues on the different HTLV-infected inducer cells (**S7B Fig**) revealed that the amount of this type of surface glycosylation was inversely correlated to the magnitude of IFN-I production by co-cultured pDCs (**Fig 6A and 6B**). As opposed, the levels of PNA lectin staining at the surface of infected cells positively correlated with their ability to transmit viral infection to target cells **(Fig 6C**). Consistent observations were obtained by confocal microscopy analysis of PNA lectin, displaying very weak PNA staining for MT2 and Hut102, the highest IFN-inducer cell lines (**S7C Fig**). Similar trend was observed for stained SBA-lectin (**S7B Fig**), thought to detect α- or β-linked *N*-acetylgalactosamine residues, albeit to with lower magnitudes of difference (**S7B Fig**), and without statistical correlation with IFN-I production (**S7D Fig**). In contrast, binding of the lectins UEIA, WGA and ConA, that recognized other glycosylated residues, did not demonstrate difference between the different cell lines (**S7B Fig**). Altogether, these results suggest that the composition of the terminal oligosaccharide residues, especially dense terminal β-galactose glycosylation, present at the surface of HTLV-1-infected cell lines might inversely govern both IFN-I response by co-cultured pDCs and viral transmission.

To further study the role of terminal β-galactose residues in IFN-I induction, we performed assay to mask such surface glycosylation by pretreating C19 cells with PNA-lectin prior contact with pDC. We controlled that the PNA concentrations used in the co-culture did not affect cell viability (**S8A Fig**). The presence of PNA-lectin at the surface of C19 cells significantly increased pDC-induced IFN-I production (**Fig 6D**). Conversely, the removal of sialic acid using neuraminidase treatment resulted in augmented exposure of terminal β-galactose at the surface of treated C91 cells (*i*.*e*., at levels similar to that of C19 cells, **Fig 6E**), and in significant decreased of pDC-induced IFN-I production (**Fig 6D**). Of note, the limited impact of neuraminidase treatment on pDC IFN-I production likely result from its short timeframe of impact on the exposure of β-galactoside residues, as revealed by the reduction overtime of PNA staining of neuraminidase-treated cells (**S8B Fig**). Thus, to strengthen the role of β-galactoside residues in the regulation of pDC IFN-I production, we determined whether viral expression in PBMCs from HAM/TSP patients was also associated with a modulation of PNA staining. *In vitro* culture of primary PBMCs from both healthy donors and HAM/TSP patients is enough to expose β-galactoside residues (**S8C and S8D Fig**). Consequently, β-galactoside was not preferentially/exclusively induced in PBMCs that expressed HTLV-1 (**S8E Fig**). However, we observed a higher proportion of infected PBMCs that expressed β-galactoside residues in the PBMCs from patient #1620 compared to the two others (**S8E Fig**). Interestingly, although the level of virus re-expression in PBMCs from patient #1620 was the highest (i.e. 70% of positive PBMCs compare to 40% for #1668 and 5% for #1485, see **S1 Fig**), this was not associated with a higher induction of type-I IFN by co-cultured with pDCs (**Fig 1I**, compare patients #1620 to #1668). Altogether, our results show that β-galactoside glycosylations at the surface of infected cells likely negatively regulate pDC activation by HTLV-infected cells.

## Discussion

Evidences suggested that IFN-I response is likely pivotal to repress HTLV-1 replication [18,19,50,51]. Yet, the persistent HTLV infection is thought to result from escape viral mechanisms and consequent failure of the immune detection and clearance of infection [52]. Along this line, HTLV-1 inhibits IFN-I induction and signaling [12–15] [16], leading to very limited production of IFN-I by infected cells. In this study, we elucidated an alternative sensing pathway mediated by the recognition of infected cells by pDCs, a sentinel cell type known to be a potent producer of IFN-I. We demonstrated that pDCs preferentially sense HTLV-1 infected cells via physical contact rather than HTLV-1 cell-free virions. This sensing pathway is thus congruent with the absence of, or very low, detection of cell-free virus in the blood of infected patients [53], a consequence of active repression of viral expression [54]. Recent report suggested that viral latency observed *in vivo* might be transiently relieved under changes in nutrients availability in the extra-cellular environment of the infected cells [55], thus potentially leading to sporadic viral expression in privileged sites such as lymphoid organs. Thus localized IFN-I response by pDCs upon contact with transiently reactivated infected cells would be in agreement with the absence of detection of IFN-I at the systemic level in infected carriers [56].

HTLV-1 viral transmission through physical contact likely compensates for the low levels of cell-free viruses found in the patients and/or their poor infectivity [35,53,57]. Upon cell contact, the virus is transmitted either through a virological synapse, in which virus assembly and budding are polarized toward the cell contact [58], or through the transfer of viral biofilm-like structures, an extracellular accumulation of viruses embedded in the infected-cell extracellular matrix (ECM) [34], both mechanisms being likely not mutually exclusive. Here, we showed that isolated viral biofilm-like structure is sufficient to trigger IFN-I production by pDCs. We further propose that the increased potential of biofilm-like structures for pDC activation, as compared to cell-free virus, could be due to components of these structures favoring the transmission of the pDC-activating signal, possibly by tethering the immunostimulatory RNA carrier to the pDC surface. Although pDCs are largely refractory to most viral infection [23], Jones *et al*. [5] observed viral production by pDCs exposed to cell-free HTLV-1 for at least 3 days. This timeframe is longer than our experimental setting (*i*.*e*., 24 hours, in accordance with previously reported pDC half-life limited to couple of days [59,60]), and during which we failed to detect productive HTLV-1 infection of pDCs. Thus, productive infection of pDCs by HTLV-1 is very unlikely needed for their rapid IFN-I production induced by contact with infected cells.

Our results suggested that pDC sensing of HTLV-infected cells is mediated via the HTLV entry receptor Glut-1, and NRP-1/BDCA-4 receptor seems dispensable. Previous report showed that infection of myeloid DC by cell-free HTLV-1 particles is independent of NRP-1/BDCA4, viral binding being ensured by the DC-SIGN lectin [61]. It is conceivable that pDC-expressed glycosylated surface factors, including lectins and HSPGs known as capture molecules for HTLV and other viruses, could act as cofactor for HTLV capture at the pDC surface via glycan-mediated interactions with HTLV before its delivery to Glut-1. Alternatively, virus delivery through cell-cell contact could bypass the need for attachment factors and virus concentration at the surface of target cells before virus interaction with its cognate receptor. Our results showed that heparin, an HSPG mimic, increased the frequency of pDC contacts with HTLV-1 infected cells and IFN-I production by pDCs in response to HTLV-1-infected cells, implying a putative function of HSPG in the stabilization of the pDC-infected cells interface required for the capture of the immunostimulatory RNA carrier present in the HTLV-1 biofilm-like structures by pDCs.

Our results uncovered differences of surface glycan pattern of HLTV-infected cells, including the composition of the terminal oligosaccharide residues at the cell surface (*i*.*e*., plasma membrane), with densification of certain residues being inversely correlated to the level of pDC IFN-I response. Further, enzymatic and pharmacological inhibition/modulation of the cell surface glycans impacted pDC response to infected cells. These observations support the proposition that the extracellular matrix and/or glycosylated proteins expressed at the plasma membrane of the infected cells likely govern both IFN-I production by co-cultured pDCs and viral transmission. Importantly, our results suggest that these two processes are inversely correlated.

By analogy to previously reported abilities of several viruses to modify ECM composition of the host cells to favor their own dissemination and/or immune escape [62–64], one might speculate that the chronic infection by HTLV modulates the cell surface glycan pattern [65]. We showed that terminal β-galactoside glycosylation density is inversely correlated with the ability of infected cells to promote contact-dependent pDC IFN-I production. Together with the absence of correlation between the levels of pDC IFN production, the amount of viral RNA production or capture of HTLV by pDCs, shown for different HTLV-infected cells, our results suggest that composition of the extracellular matrix and/or cell surface expression of glycosylated proteins that embed cell surface-attached viruses of the different HTLV-infected cells might regulate pDC activation. The results obtained using PBMCs from HAM/TSP patients suggest that β-galactoside residues induction at the surface of infected cells might not however be regulated by viral expression. In addition, we could not determine whether these residues are specific components of the viral biofilm, or whether β-galactoside-containing proteins surrounding the viral biofilm at the plasma membrane of the infected cells are enough to regulate IFN-I production.

Previous reports showed that pDC response to viral infections can be modulated by different cell surface factors including the regulatory receptors ILT7, BDCA2 or DCIR [66,67]. For example, ILT7 binds BST-2, an IFN-induced gene, initially described as an HIV restriction factor that impedes viral release from infected cells [68]. Since HIV Vpu protein counteracts viral tethering by BST2, virus-free surface BST-2 can readily interact with ILT7, and thereby inhibits pDC IFN-I production [69]. While BST-2 is also expressed upon HTLV-1 infection, as opposed to the negative regulation of HIV release, it participates in efficient HTLV-1 cell-cell transmission [70]. Other negative regulatory receptors e.g., BDCA2 and DCIR bind complex-type sugars chains (terminal β-galactoside containing complex sugars [71,72], and mannose/fucose containing complex sugars [73,74], respectively). Since pDC IFN-I response to HTLV is modulated by the available terminal sugar composition at the surface of infected cells, one might speculate that the density of specific glycans, via the interaction with negative regulators, *e*.*g*., BDCA2 might regulate the levels of pDC IFN-I production induced by HTLV-infected cells as previously shown in other contexts [75–77]. Interestingly, these dense specific glycans might not be part of the biofilm, but still might be engaged after contact with the pDC. Thus, along with the interaction of the viral envelop protein with the Glut-1 receptor, other cell surface factors, including heparan sulfate-containing proteins, and terminal galactoside-conjugated proteoglycans, at the pDC/infected-cell interface, could regulate the strength of pDC activation. Altogether our results provided an original illustration of the regulation of pDC IFN-I response by the surface glycan pattern of infected cells.

## Materials and methods

### Cells

Jurkat cells (from ATCC, ref ACC 282) stably transfected with a plasmid encoding the luciferase (Luc) gene under the control of the HTLV-1 long terminal repeat (LTR) promoter and the HTLV-1 Tax-transactivator (Jurkat-LTR-Luc) [34] were maintained under hygromycin selection (450 μg/mL, Sigma) in culture RPMI medium: RPMI 1640 medium supplemented with 10% fetal calf serum (FCS; Gibco Life Technologies), L-Glutamine (2 mM, Gibco Life technologies) and penicillin-streptomycin (100 U/mL and 100 μg/mL respectively; Gibco Life Technologies). C91-PL (HTLV-1 infected T-cell line, Cellosaurus, ref CVCL_0197), MT-2 (HTLV-1 infected T-cell line, NIH, ref 237 and [78]), Hut102 (HTLV-1 infected T-cell line, Cellosaurus, ref CVCL_3526 and [79]) and C8166 (HTLV-1 infected T-cell line which does not produce infectious virus [39], ECACC ref 88051601) were maintained in culture RPMI medium. PBMCs from healthy blood donors or from HAM/TSP patients were cultured 18h in RPMI medium supplemented with 20% FCS supplemented with IL-2 (150 U/mL) and PHA (1μg/mL). C19 (HTLV-2 infected cell line, obtained from [80]) and MO (HTLV-2 infected cell line, ATCC ref CRL-8066) were maintained in culture RPMI medium supplemented with 20% FCS. The human fibrosarcoma cell line containing a plasmid encoding the luciferase gene under the control of the immediate early IFN-I inducible 6–16 promoter (HL116) (a kind gift from S. Pelligrini, Institut Pasteur, France) [81] was maintained under HAT selection in DMEM medium supplemented with 10% FCS and penicillin-streptomycin (100 U/mL and 100 μg/mL respectively). All cells were grown at 37°C in 5% CO_2_.

### Reagents

Ficoll-Hypaque (GE Healthcare Life Sciences); LPS, TLR7 agonist (R848) and TLR9 agonist (ODN2216) (Invivogen); TLR7 antagonist, IRS661 (5’-TGCTTGCAAGCTTGCAAGCA-3’) synthesized on a phosphorothionate backbone (MWG Biotech); Fc Blocking solution (MACS Miltenyi Biotec); BDCA-4-magnetic beads for selective isolation of pDCs (MACS Miltenyi Biotec); IL-6 ELISA kit (Affymetrix, eBioscience); Lipofectamine 2000 (Life Technologies); 96-well format transwell chambers (Corning); LabTek II Chamber Slide System, 96-Well Optical-Bottom Plates and Nunc UpCell 96F Microwell Plate (Thermo Fisher Scientific); Vibrant cell-labeling solution (CM-DiI, Life Technologies); rat anti-HSPG antibody (clone A7L6, Upstate Biotechnology) Hoescht and anti-mouse AlexaFluor 647-conjugated secondary antibody (Life Technologies); anti-mouse DyLight 488-conjugated secondary antibody (Vector); anti-rat APC-conjugated secondary antibody (SouthernBiotech); High Capacity cDNA reverse transcription kit (Applied Biosystems); Powerup Sybr Green Master Mix (Applied Biosystems); pDC specific markers: mouse PE or APC-conjugated anti-CD123 (clone AC145, Miltenyi), mouse APC-conjugated anti-BDCA-2 (AC144; Miltenyi); mouse PE-conjugated anti-TRAIL (ThermoFisher); Metalloproteinase 9 (Enzo Life Sciences); FITC-conjugated Peanut Agglutinin (PNA) (Sigma Aldrich); Alexa Fluor 680-conjugated Wheat Germ Agglutinin (WGA) (Thermo Fisher); FITC-conjugated WGA, Soy bean Agglutinin (SBA), Ulex europaeus agglutinin I (UEA-I) and Concanavalin A (ConA) were from Vectors; poly-L-lysine (Sigma, P4832), anti-HTLV-1 p19^gag^ antibody (1:1000, Zeptometrix); recombinant VEGF_165_ protein (R&D); Glut-1.RBD.GFP (Metafora biosystem); recombinant IFN-α 2a (TEBU BIO PBL); anti-HTLV-1 Env gp46 antibody (1:1000, Zeptometrix), luciferase reporter activity assay (Promega); paraformaldehyde 20% (PFA; Electron Microscopy Sciences); saponin (Sigma); Heparin (Sigma). HTLV-1 molecular clone (pACH) and HTLV-1 molecular clone lacking the expression of the envelope protein (pACH ΔEnv, [82]) were provided by Dr. Pique (Institut Cochin, France). The Alexa Fluor 488-conjugated anti-Tax antibody (LT-4) was provided by Pr Tanaka (University of Ryukyus, Japan).

### Ethics statement

The pDCs and PBMCs were isolated from blood or cytapheresis units from healthy adult human volunteers which was obtained according to procedures approved by the “Etablissement Français du sang” (EFS) Committee. All donors provided informed consent to EFS. PBMCs from HAM/TSP patients were obtained in the context of a Biomedical Research Program approved by the Committee for the Protection of Persons, Ile-de-France II, Paris (2012-10-04 SC). All individuals gave informed consent.

### Isolation of pDCs and *ex vivo* co-culture experiments

PBMCs were isolated using Ficoll-Hypaque density centrifugation. pDCs were positively selected from PBMCs using BDCA-4-magnetic beads (MACS Miltenyi Biotec) and pDCs were depleted from PBMCs, as previously described [24,25]. The typical yields of PBMCs and pDCs were 800x10^6^ and 2x10^6^ cells, respectively, with a typical purity of >91% pDCs, as we previously reported [24,25].

After isolation, pDCs (2x10^4^) were platted in 96-well round bottom plates and cultured at 37°C with HTLV-1 or HTLV-2 infected cell lines (2x10^4^ or other count when indicated), or with PBMCs from healthy donors or from HAM/TSP patients (2x10^4^), or with Jurkat cells microporated with the HTLV-1 molecular clone pACH or pACH ΔEnv (2x10^4^), or with Jurkat cells (2x10^4^) as negative control, or with isolated HTLV-1 biofilm-like structures (100μL), or with HTLV-1 biofilm-like structures depleted cells (2x10^4^). When indicated, HTLV-2 infected cells (C19, 10^6^ cells in RPMI culture medium) or HTLV-1 infected cells (C91, 10^6^ in PBS) were treated with PNA (10 μg/ml, SIGMA) for 30 min at 4°C or neuraminidase (0,1U/ml, SIGMA) for 1h at 37°C respectively. Treated cells were then washed twice in RPMI culture medium prior to co-culture (2×10^4^) with pDC (2×10^4^). Culture with isolated pDCs or PBMCs were maintained in RPMI 1640 medium (Life Technologies) supplemented with 10% FCS, 10 mM HEPES, 100 units/mL penicillin, 100 μg/mL streptomycin, 2 mM L-glutamine, non-essential amino acids and 1 mM sodium pyruvate at 37°C/5% CO_2_. The supernatants were collected at 20-24h after co-culture. When indicated, infected cells or uninfected cells were co-cultured with pDCs in 96-well format transwell chambers separated by a 0.4 mm membrane (Corning), as previously [24,25].

### IFN-α/β and IL-6 detection

HL116 cells were seeded at 2.10^4^ cells/well in 96-well plate 24 h prior the assay, and incubated for 17 h with supernatant collected from pDC co-cultures (100 μL) or serial dilution of recombinant human IFN-α 2a (PBL Interferon Source), used for standard curve titration. Cells were then lysed and luciferase activity assayed. IFN-I levels were expressed as equivalent of IFN-α 2a concentration, in Unit/mL. The detection of IL-6 by ELISA was performed as previously[24] using kit (Affymetrix, eBioscience) and according to the manufacturer instructions.

### Jurkat cells microporation

Jurkat cells (8x10^4^ cells) were transfected with 3 μg of pACH or pACH ΔEnv together with 1 μg of pSG5M-Tax1 [83] using the Neon Transfection System (ThermoFischer Scientific) following manufacter’s instructions. Cells were cultured 48h at 37°C before co-culture with pDCs.

### Isolation of viral biofilm-like structures and purification of cell-free viruses

HTLV-1 viral biofilm-like structure was prepared with a method that is slightly different from the original one [34] and as previously described [35]. Briefly C91-PL cells were platted (3x10^5^ cells/mL) and cultured for 4 days. HTLV-1–infected cells were washed twice in RPMI-1640 serum-free medium and incubated at 1x10^6^ cells/ml for 1 h at 37°C, with gentle agitation every 10 minutes. Then, FCS (10% final) and penicillin-streptomycin (100 μg/mL final) were added, and cells centrifuged. Supernatant containing biofilm-like structures preparation was collected and supplemented with Hepes (10 mM), non-essential amino acid (2.5 mM), sodium pyruvate (1 mM), β-mercaptoethanol (0.05 mM) before immediate use.

Cell-free viruses were also obtained from C91-PL cells (10^6^ cells/mL) cultured for 24h at 37°C 5% CO_2_. Supernatant were clarified by centrifugation (5 minutes at 800g) and filtrated through a 0.45 μm-diameter pore filter (Millipore, MA) to eliminate cell debris. Virions were purified by ultracentrifugation through a 20% (wt/vol) sucrose cushion at 100,000*g* in SW32 (Beckman) for 1h30 at 4°C and stored at -80°C before use. Virus concentration was determined using Retrotek HTLV-1/2 p19^gag^ Antigen ELISA kit (Zeptometrix) following manufacturer’s instructions and as previously described [19].

### HTLV-1 viral Biofilm-like structures shedding

C91-PL cells (10^6^ cells /mL) were treated with Metalloproteinase 9 (20 nM) in RPMI serum-free medium for 1h at 37°C 5% CO_2_. Cells were washed twice with culture RPMI medium, and immediately used. The efficacy of HTLV-1 viral biofilm-like structures shedding was controlled by analyzing gp46 viral envelope level by FACS and viral transmission to T-cells using Jurkat LTR-Luc reporter cells.

### Viral transmission to T-cells

HTLV-1 (C91-PL, Hut102, MT-2), HTLV-2 (C19, MO) or uninfected (Jurkat) cell lines (10^3^, 10^4^ or 10^5^) were co-cultured with Jurkat LTR-Luc cells (10^4^). Different ratio of infected cells/target cells (1/10; 1/1 or 10/1) were incubated for 24 hours in round-bottom 96-wells plates at 37°C. Cells were washed once with PBS and stored at -80°C as dry pellets until assayed for luciferase reporter activity using manufacturer’s instructions (Promega). Luciferase results were normalized according to the amount of proteins determined by Bradford (Biorad).

### HTLV receptor competition assay

Jurkat LTR-Luc cells (2x10^5^) were incubated in culture RPMI medium with VEGF_165_ (80–100 ng/mL) or 10μL Glut-1.RBD.GFP at 4°C during 30 minutes before co-culture with C91-PL cells (2x10^4^) or with cell-free viruses (50 ng/mL of p19^gag^ equivalent as measured by ELISA) for 2 hours at 37°C. Cells were then harvested, washed with PBS, fixed in 4% PFA, permeabilized in PBS / 1% BSA / 0.05% Saponin and stained with an anti-p19^gag^ antibody (1:1000) followed by FITC or Alexa Fluor 549-conjugated anti-mouse antibody. Fluorescence was acquired on at least 10 000 events with a FACSCanto II cytometer (BD Biosciences) and data analyzed on FlowJo software (Tree Star, Inc. Ashland, OR).

### Surface staining of Env gp46, BDCA-2/CD123, HTLV-1 receptor or lectins analyzed by FACS

HTLV-1 (C91-PL, Hut102, MT-2), HTLV-2 (C19, MO) or uninfected (Jurkat) cell lines (2x10^5^) were fixed with 4% PFA and stained with FITC-conjugated lectins (10 μg/ml) for 30 minutes at 4°C. The level of Env gp46 surface expression was determined on unfixed C91-PL cells or on Jurkat transfected cells using anti-HTLV-1 Env gp46 antibody (1:1000 in PBS-1% BSA) for 1h at 4°C followed by Alexa488-coupled anti-mouse antibody for 30 min at 4°C. pDC were surface-stained with mouse PE or APC-conjugated anti-CD123 and mouse APC-conjugated anti-BDCA-2, with Glu1.RBD.GFP protein (5μl/ 1x10^5^ cells, Metafora), or with anti-NRP-1 (clone 12C2, Biolegend). Alternatively, pDCs were fixed in 4% PFA and stained with anti HSPG antibody (1:100) for 30 min at 4°C followed by APC-conjugated anti rat antibody (1:100) for 30 min at 4°C. Cells were then washed with PBS and fluorescence acquired using 20 000 events on a FACSCanto II cytometer, and analyzed with FlowJo software (Tree Star, Inc. Ashland, OR).

### Intracellular staining of p19^gag^

Transfected Jurkat cells or PBMCs from HAM/TSP patients were cultured 18h in presence or not of IL-2 and PHA and fixed with 4% PFA, permeabilized in PBS / 1% BSA / 0.05% Saponin, and stained with anti-p19^gag^ antibody (1:1000) for 30 min at 4°C followed by DyLight488-conjugated anti-mouse antibody (1:1000). Cells were then washed and fluorescence acquired using at least 10 000 events on a FACSCanto II cytometer (BD Biosciences), and analyzed with FlowJo software.

### TRAIL expression on pDCs after coculture with HTLV-infected cells

After 24h co-culture with HTLV-1/2 infected cells, pDCs were collected, washed and stained with PE-conjugated anti-TRAIL and APC-conjugated anti-BDCA-2 antibodies. Cells were then washed and fixed in 4% PFA. Fluorescence was acquired using at least 10 000 events with a FACSCanto II cytometer (BD Biosciences) and analyzed with FlowJo.

### Viral binding, and viral capture by pDCs after co-culture with HTLV-1 infected cells

pDCs (10^5^) were co-cultured with C91-PL (HTLV-1 infected cells, 10^5^) in the presence or not of Glu1.RBD.GFP (10μl) for 4h. Cells were then washed in PBS. For subsequent viral binding analyses, cells were surface-stained with anti-gp46 antibody (1:1000) followed by Alexa Fluor 647-conjugated anti-mouse antibody (1:1000). For viral capture analyses, cells were fixed in 4% PFA, permeabilized in PBS / 1% BSA / 0.05% Saponin, and stained with anti-p19^gag^ antibody (1:1000) followed by Alexa Fluor 647-conjugated anti-mouse antibody (1:1000). After washing, pDCs were surface-stained with anti-CD123-Vioblue-conjugated antibody and fixed in 4% PFA.

### pDCs and MDDCs infection

pDCs (10^5^) or MDDCs (2.5x10^5^) were co-culture with C91-PL (10^5^) for 24h or 72h respectively. For pDCs infection analysis, cells were washed, surface-stained with Vioblue-conjugated anti-CD123 antibody, fixed and permeabilized according to the manufacturer’s instructions (eBiosciences). For MDDCs infection analysis, cells were washed in PBS and in normal goat serum (7%, Sigma), fixed and permeabilized according to the manufacturer’s instructions (eBiosciences). pDCs or MDDCs were stained with biotin-coupled anti-Tax antibody (LT4) followed by streptavidin labeled with PE-Cy7 (BioLegend, Ozyme). After extensive washes, MDDCs were finally surface-stained with a V450-coupled anti-CD11c antibody. Fluorescence was acquired using at least 10 000 events with a FACSCanto II cytometer and data analyzed on FlowJo software.

### Measurement of intracellular and secreted HTLV-1/2 particles by quantitative reverse transcription-PCR (qRT-PCR) analysis

RNAs were isolated from samples harvested in guanidinium thiocyanate citrate buffer (GTC; Sigma-Aldrich) by phenol/chloroform extraction procedure as previously described [25]. Reverse transcription was performed using the random hexamer-primed High Capacity cDNA reverse transcription kit (Applied Biosystems) and quantitative PCR was carried out using the Powerup SYBR Green Master Mix (Applied Biosystems). The absolute numbers of HTLV-1 transcripts were normalized to the total amount of RNA. For supernatant samples, qRT-PCR was controlled by the addition of exogenous carrier RNAs encoding *xef1α* (xenopus transcription factor 1α) in supernatant diluted in GTC buffer, as previously described [24,25].

For quantification of viral genomic RNA the following primers were used: HTLV-1 Forward (AAAGCGTGGAGACAGTTCAGG), HTLV-1 Reverse (CAAAGGCCCGGTCTCGAC), HTLV-2 Forward (CCTTGGGGATCCATCCTCTC), HTLV-2 Reverse (TCTCTAAAGACCCTCGGGGAG). For quantification of viral RNA present in the supernatant of infected cells, the following primers were used: Tax 1 Forward (GGATACCCAGTCTACGTGTTTGG), Tax 2 Forward (GGATACCCCGTCTACGTGTTTGG), Tax 1/2 Reverse (GGGGTAAGGACCTTGAGGGT).

### Imaging combined with flow cytometry analysis of pDC/infected cell conjugates using Image Stream X technology

HTLV-1 (C91-PL, Hut102, MT-2), HTLV-2 (C19, MO) cell lines and control uninfected Jurkat cells (5x10^5^) were transduced in 24 well plate with lentiviral-based vector pseudotyped with VSV glycoprotein to stably express GFP. Briefly, 10^5^ GFP-expressing HTLV infected cells and control cells were co-cultured with 4x10^4^ pDCs in low-adherence micro-plate designed for cell harvesting by temperature reduction (Nunc UpCell 96F Microwell Plate from Thermo Scientific) for 4–5 hours at 37°C, and, as indicated, in presence or not of heparin. The co-cultured cells were detached by 20 minute-incubation at room temperature, harvested and fixed in 4% PFA. Cells were then washed twice with staining buffer (PBS 2% FBS), and pDCs were stained by the pDC-specific CD123 marker. Co-cultured cells were analyzed by Image Stream X technology (Amnis) at magnification x40 using IDEAS software, as previously described [24,25]. The cell population defined as pDC/HTLV-infected or uninfected cell conjugates comprises conjugates of at least one CD123 APC positive cell and at least one GFP positive cell among the total of CD123 APC-positive cells, GFP-positive cells and conjugates. As shown by representative images (**S4A Fig**), the population gated as pDC-HTLV-1-infected cell conjugates corresponded to GFP positive/CD123 positive cell conjugates, as expected, with 80–95% purity. The cell populations were sorted using masks (IDEAS software) to eliminate cells out of focus and/or with saturating fluorescent signal, and then selected based on cell size of the positive cells (*i*.*e*., fluorescent signal area).

### Immunofluorescence staining

For lectin/HTLV-1/2 virus localization analysis, HTLV-1 (C91-PL, Hut102, MT-2), HTLV-2 (C19, MO) or uninfected (Jurkat) cell lines cultured on Lab-tek chamber slides (Nunc) previously treated with 0.01% poly-L-lysine (Sigma, P4832) were surface-stained with FITC-conjugated PNA or WGA (10μg/ml), fixed in 4% PFA, then permeabilized and stained with antibodies against HTLV-1/2 matrix protein p19^gag^ (1:1000) followed by Alexa Fluor549-conjugated anti mouse antibodies. Cells were counterstained with DAPI-Fluoromount G before analysis on Zeiss LSM 800 microscope. Images were acquired on ImageJ.

For pDC/HTLV-infected cells conjugates analysis, 4x10^4^ pDCs were stained using 0.5 μM Vibrant cell-labeling solution as previously[24]. Labeled pDCs were washed twice with PBS and then co-cultured with 2x10^4^ HTLV infected cells for 4–5 hours at 37°C, in a 96-well optical-bottom plate pre-coated with 8 μg/mL poly-L-lysin for 30 minutes at 37°C. Cells were then fixed in 4% PFA, washed with PBS and PBS-3%BSA, and stained with anti-HTLV-1 Env gp46 antibody (1:1000 in PBS—3%BSA) for one hour at room temperature. Prior antibody staining, cells were stained by WGA lectin coupled to Alexa 680 (Molecular Probes, ref W32465) diluted at 10μg/mL in HBSS, for 10 minutes at room temperature, then washed three times with HBSS. After three washes with PBS, cells were incubated with Alexa488-conjugated-anti-mouse antibody in 3% BSA-PBS and added to the cells along with Hoechst diluted at 1:500 (Molecular Probes) for 1 hour at room temperature. After three washes with PBS, cells were observed with a Zeiss LSM 710 laser scanning confocal microscope. The quantification of the phenotypes defined to as clusters at the contact were performed using Image J software package.

### Statistical analysis

Statistical analysis was performed using PRISM v7.03 software (Graphpad). One-way analysis of variance (ANOVA) with Sidak’s multiple comparison test was used to determine statistically significant differences. Paired two-tail t-test was used to compare two groups from the same donor. Differences were considered significant if the p-value was < 0.05.

## Supporting information

S1 Fig Viral expression in Jurkat cells transfected with the molecular clones or in PBMCs obtained from HAM/TSP patients before or after culture in presence of IL2 and PHA. **A.** Expression of Env gp46 viral protein (left histograms) or p19^gag^ (right histograms) was determined by flow cytometry 48 h after transfection of Jurkat cells with Tax plasmid alone or with Tax plasmid and the molecular clone (pACH) or with Tax plasmid and a molecular clone lacking the envelope glycoprotein expression (pACH-ΔEnv) (mean ± SD; 3 independent experiments, and one representative histogram for each staining is shown on the right.). Asterisks indicate statistically significant differences calculated using ANOVA followed by Sidak’s multiple comparison test: *** p<0.001; ns = non significant. **B.** Viral expression as determined by p19^gag^ detection in PBMCs from 3 independent HAM/TSP patients before (white histograms) and after (grey histograms) 18h of *in vitro* culture in presence of IL2 and PHA.
(TIF)

S2 Fig Viral infection of pDCs or MDDCs and viral binding with or without competition using RBD or VEGF165. **A.** pDCs or MDDCs were co-cultured with HTLV-1 infected cells (C91-PL) or control Jurkat cells (cont) for 24h or 72h respectively. Productive viral infection was measured by flow cytometry using intracellular Tax detection in the CD123+ pDC population or in the CD11c+ MDDC population. CD123 negative or CD11c negative population identified the C91-PL cells present in the coculture. Representative of 3 independent experiments. **B.** pDCs were co-cultured with HTLV-1 infected cells (C91-PL) for 4h in presence (grey histogram) or not (white dot line histogram) of Glut-1.RBD.GFP (RBD) and viral binding on pDCs was measured by flow cytometry using Env gp46 staining in the CD123+ pDC population. Representative of 3 independent experiments. **C.** FACS gating strategy used for the analysis of VEGF_165_ competition. Cell populations (C91-PL; Jurkat cells or co-culture of C91-PL and Jurkat cells) were gated based on their size (FSC) and granulosity (SSC), and p19^gag^ expression determined on each population. C91-PL population was used as a positive control for p19^gag^ expression while Jurkat cell population was used as a negative control. The percentage of p19^gag^ positive Jurkat cells in the co-culture with C91-PL is shown. (Representative of 3 independent experiments.).
(TIF)

S3 Fig Biofilm depletion decreased both pDC IFN-I production and viral transmission. **A.** IFN-I amount as determined in Fig 3F. **B.** Infectivity levels, determined as in Fig 3G. **A-B.** Results are expressed as percentages relative to untreated co-cultures (mean ± SD; 3 independent experiments). Asterisks indicate statistically significant differences calculated using t-test: * p<0.05; ns = non significant.
(TIF)

S4 Fig Increase of pDC IFN-I production and cell contact by heparin treatment. **A.** Imaging flow cytometry analysis (ImageStream) of HTLV-1 infected cells, which stably express GFP, and co-cultured with pDCs for 4–5 hours, as in the Fig 4A. pDCs are detected by the immunostaining of CD123, a pDC specific marker. Representative pictures of the cell population gated as conjugates between pDCs and GFP expressing infected cells (upper panels), of the cell population gated as HTLV-1 infected cells (GFP positive cells, middle panels) and of the cell population gated as pDCs, single cells (CD123 positive cells, lower panels), are shown. Panels, as displayed from the left to the right, Bright field; GFP field; APC field; GFP/APC field and Merge. **B.** Quantification of the effect of heparin treatment (as in Fig 4B) on IFN-I production in SNs of pDCs co-cultured with HTLV-1-infected cells or HTLV-1-purified biofilm-like structure normalized to the amount of p19 measured in each biofilm-like structures preparation. The results are expressed as fold-increase relative to the untreated controls (mean ± SD; 10 and 3 independent experiments for HTLV-1 infected cells and biofilm-like structure, respectively). Asterisks indicate statistically significant differences calculated using ANOVA followed by Sidak’s multiple comparison test: *** p<0.001.
(TIF)

S5 Fig Lack of correlation between pDC-induced IFN-I production and HTLV RNA production or cell-conjugates formation. **A-C.** IFN-I amounts (U/ml) induced by HTLV- infected cells plotted against the corresponding intracellular RNA levels (A), extracellular RNA levels (B) or the percentage of cell-conjugates (C). Compute correlation p values are indicated**. D.** Infectivity levels determined after co-culture of Jurkat-LTR-Luc reporter cells (10^4^ or 10^5^) with HTLV-1 or HTLV-2 infected cells (10^4^ or 10^5^). The infected cells/reporter cell ratio (1:10 represents 10^4^ infected cells for 10^5^ reporter cells, 1:1 represents 10^5^ infected cells for 10^5^ reporter cells, 10:1 represents 10^5^ infected cells for 10^4^ reporter cells) is indicated on the right of the graph. RLU, relative light unit. Arrows indicate the maximum level of RLU relative to viral transmission for each cell line setting. (mean of 3 independent experiments).
(TIF)

S6 Fig Viral accumulation at the surface of HTLV-infected cells and IFN-I induction by HTLV-2 infected cells, as that induced by HTLV-1 infected cells, requires TLR7 signaling and receptors for viral fusion but not for viral binding. **A and C.** Impact of Glut-1 binding competitor (RBD, 5μL/10^5^ cells, A) or NRP-1/BDCA-4 binding competitor (VEGF_165_, 100 ng/mL, C) on IFN-I activity in SNs of pDCs co-cultured with HTLV-1-infected cells (C91-PL) or HTLV-2 infected cells (C19). **B and D** Corresponding infectivity levels, determined as in Fig 2D. The results are expressed as percentages relative to untreated co-cultures (mean ± SD; 3–5 independent experiments). Asterisks indicate statistically significant differences calculated using ANOVA followed by Sidak’s multiple comparison test: **** p<0.0001; ns = non significant. **E.** Quantification of IFN-I activity in pDCs SNs. Cells were pre-incubated, or not, with TLR7 inhibitor (IRS661, 0.35 μM), as indicated, then co-cultured with infected cells HTLV-2 cells (C19 cells) or with control cells and stimulated by agonist of TLR7 (R848, 50 ng/mL) or of TLR9 (ODN2216, 0.1 μM). Results are expressed as percentages relative to IFN-I activity determined in the absence of TLR7 inhibitor, set at 100 (means ± SD; n = 3). **F.** Representative images (of 3 independent experiments) obtained by confocal microscopy of HTLV-1 (C91-PL; MT-2 or Hut102) or HTLV-2 (C19 or MO) -infected cells immunostained against p19^gag^ (red) and counterstained with DAPI for nuclei (blue). Scale bar = 5μm.
(TIF)

S7 Fig Surface binding of lectins on HTLV-1/2 infected cells. **A.** Name and binding specificity of the different lectins. **B.** Surface binding quantification of the various lectins on the HTLV-1/2 infected cells determined by FACS using FITC-coupled lectins. (Mean ± SD of 3 independent experiments). Asterisks indicate statistically significant differences calculated using ANOVA followed by Sidak’s multiple comparison test: * p<0.05; ** p<0.01; **** p<0.0001; ns = non significant **C.** Representative images obtained by confocal microscopy of HTLV-1- (C91-PL; MT-2 or Hut102) or HTLV-2- (C19 or MO) infected cells immunostained with FITC-coupled PNA (green) and DAPI for nuclei (blue). Scale bar = 5μm. **D.** Correlation curve of SBA expression (MFI) and pDC IFN-I production induced by coculture with HTLV-1 and HTLV-2 infected cells. Compute correlation p value is indicated.
(TIF)

S8 Fig Surface expression of β-galactoside glycan on PBMCs from healthy or HAM/TSP patients. **A.** The percentage of living cells (*i*.*e*., aqua negative as measured by FACS using live-dead Aqua reagents from Thermofisher) was determined on C19 cells treated or not with PNA (10μg/ml) for 30 minutes (means ± SD; n = 5). **B.** C91-PL cells were treated (filled histograms) or not (unfilled histograms) with Neuraminidase (Neu, 0.1 U/ml) for 1h and were either immediately stained with PNA (grey histograms) or stained after 24h *in vitro* culture (blue histograms). Representative of 3 independent experiments. **C.** PBMCs from 3 independent healthy donors were stained with PNA before (grey histograms) or after 18h *in vitro* culture in presence of IL2 and PHA (white histograms). The percentage of PNA positive PBMCs is indicated on the right of each histograms. Representative of 3 independent experiments. **D.** PBMCs from 3 independent HAM/TSP patients were stained with PNA before (grey histograms) or after 18h *in vitro* culture in presence of IL2 and PHA (white histograms). The percentage of PNA stained PBMCs is indicated on each histogram. Representative of 3 independent experiments. **E.** PBMCs from 3 independent HAM/TSP patients were cultured for 18h *in vitro* in presence of IL2 and PHA. Viral expression and β-galactoside residues levels by co-staining with p19^gag^ and PNA were determined by flow cytometry. The percentages of p19^gag^ and PNA double-positive PBMCs are indicated on each plot. Representative of 3 independent experiments.
(TIF)
