# Supplementary figures and images for: Sensing of cell-associated HTLV by plasmacytoid dendritic cells is regulated by dense β-galactoside glycosylation
Source: PLoS Pathog. 2019 Feb 28;15(2):e1007589. doi: 10.1371/journal.ppat.1007589 (PMC6413949; doi:10.1371/journal.ppat.1007589)

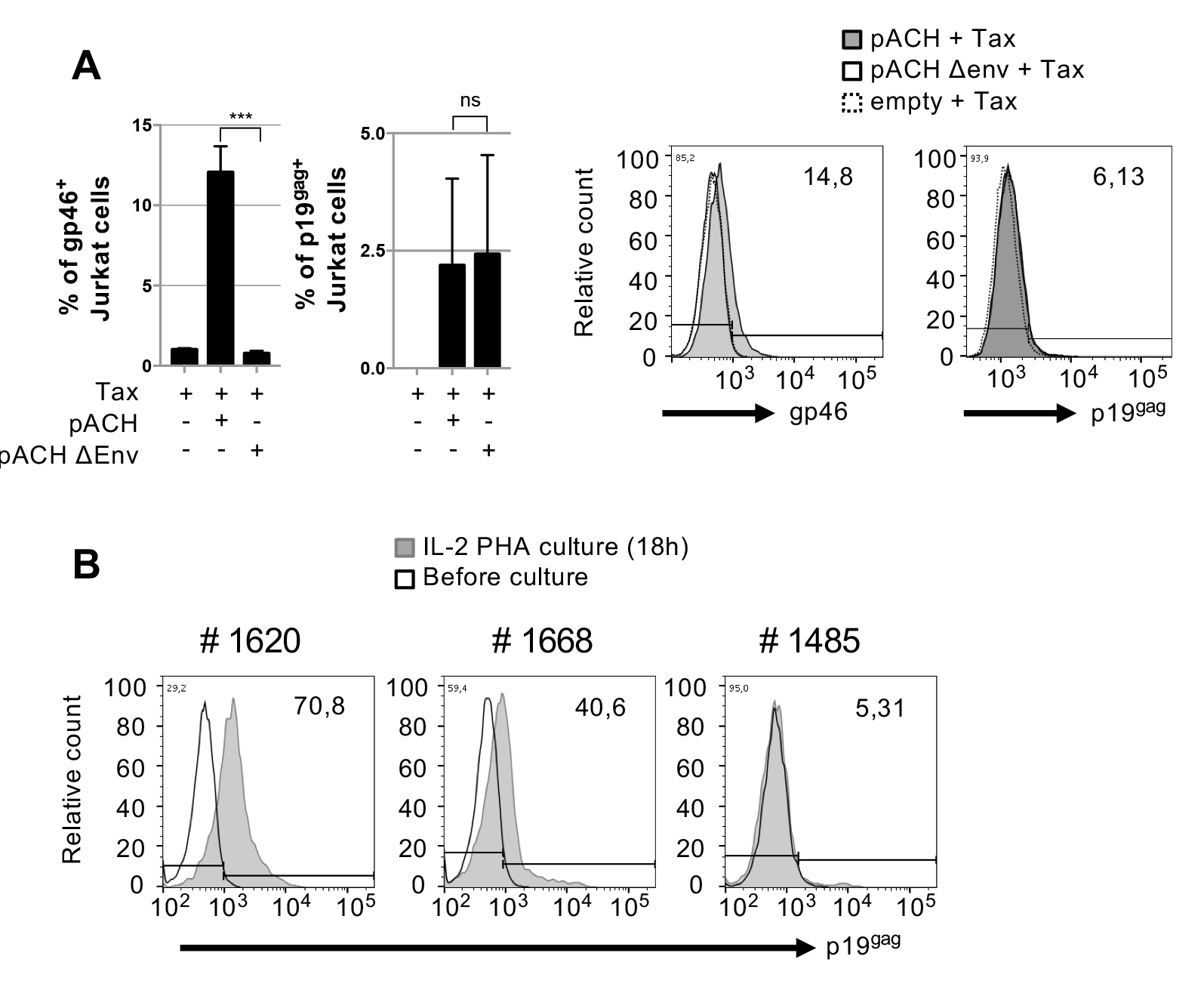

Supplement: S1 Fig — A. Expression of Env gp46 viral protein (left histograms) or p19gag (right histograms) was determined by flow cytometry 48 h after transfection of Jurkat cells with Tax plasmid alone or with Tax plasmid and the molecular clone (pACH) or with Tax plasmid and a molecular clone lacking the envelope glycoprotein expression (pACH-ΔEnv) (mean ± SD; 3 independent experiments, and one representative histogram for each staining is shown on the right.). Asterisks indicate statistically significant differences calculated using ANOVA followed by Sidak’s multiple comparison test: *** p<0.001; ns = non significant. B. Viral expression as determined by p19gag detection in PBMCs from 3 independent HAM/TSP patients before (white histograms) and after (grey histograms) 18h of in vitro culture in presence of IL2 and PHA. (TIF) [file ppat.1007589.s001.tif]

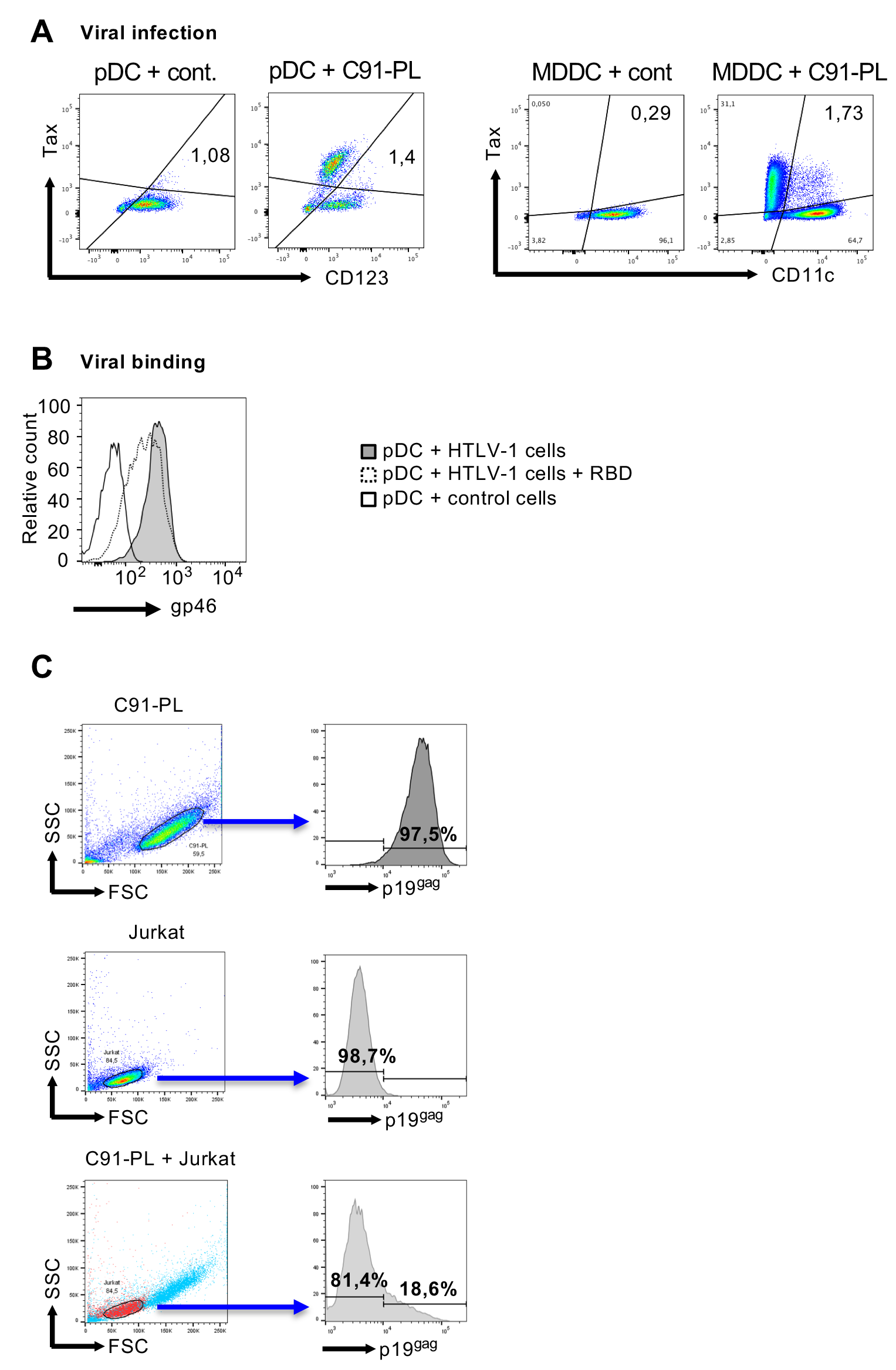

Supplement: S2 Fig — A. pDCs or MDDCs were co-cultured with HTLV-1 infected cells (C91-PL) or control Jurkat cells (cont) for 24h or 72h respectively. Productive viral infection was measured by flow cytometry using intracellular Tax detection in the CD123+ pDC population or in the CD11c+ MDDC population. CD123 negative or CD11c negative population identified the C91-PL cells present in the coculture. Representative of 3 independent experiments. B. pDCs were co-cultured with HTLV-1 infected cells (C91-PL) for 4h in presence (grey histogram) or not (white dot line histogram) of Glut-1.RBD.GFP (RBD) and viral binding on pDCs was measured by flow cytometry using Env gp46 staining in the CD123+ pDC population. Representative of 3 independent experiments. C. FACS gating strategy used for the analysis of VEGF165 competition. Cell populations (C91-PL; Jurkat cells or co-culture of C91-PL and Jurkat cells) were gated based on their size (FSC) and granulosity (SSC), and p19gag expression determined on each population. C91-PL population was used as a positive control for p19gag expression while Jurkat cell population was used as a negative control. The percentage of p19gag positive Jurkat cells in the co-culture with C91-PL is shown. (Representative of 3 independent experiments.). (TIF) [file ppat.1007589.s002.tif]

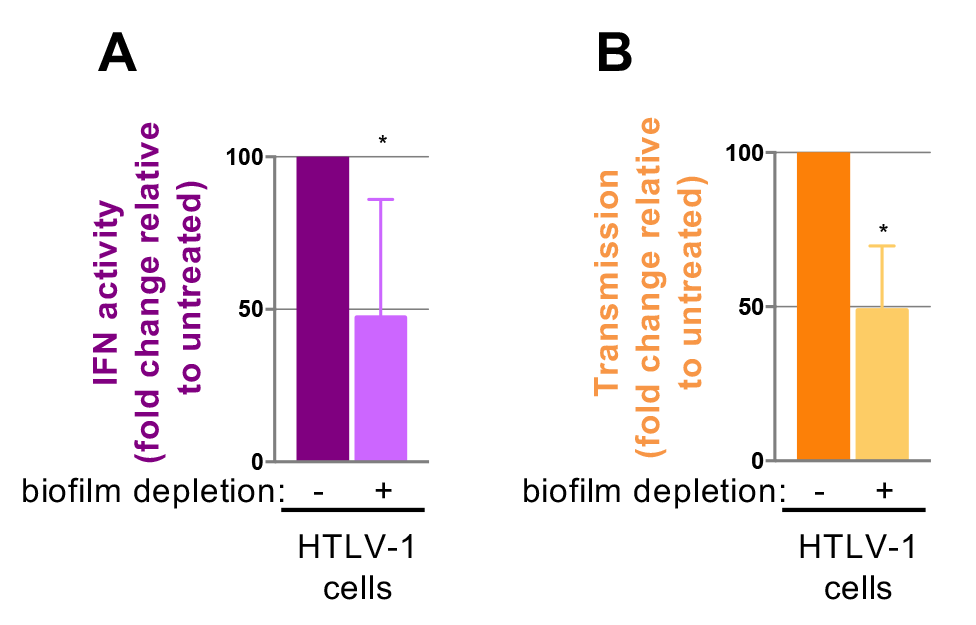

Supplement: S3 Fig — A. IFN-I amount as determined in Fig 3F. B. Infectivity levels, determined as in Fig 3G. A-B. Results are expressed as percentages relative to untreated co-cultures (mean ± SD; 3 independent experiments). Asterisks indicate statistically significant differences calculated using t-test: * p<0.05; ns = non significant. (TIF) [file ppat.1007589.s003.tif]

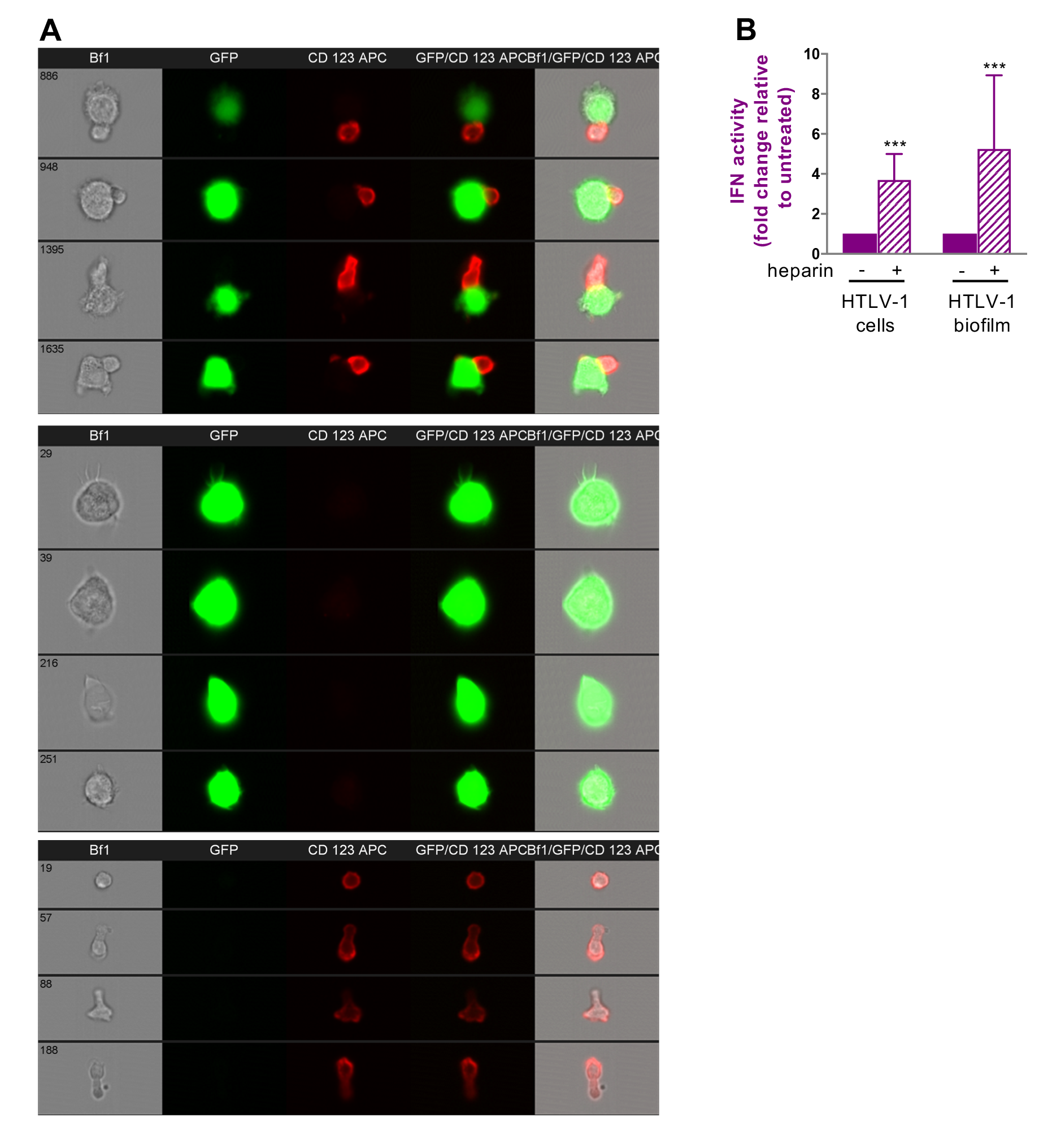

Supplement: S4 Fig — A. Imaging flow cytometry analysis (ImageStream) of HTLV-1 infected cells, which stably express GFP, and co-cultured with pDCs for 4–5 hours, as in the Fig 4A. pDCs are detected by the immunostaining of CD123, a pDC specific marker. Representative pictures of the cell population gated as conjugates between pDCs and GFP expressing infected cells (upper panels), of the cell population gated as HTLV-1 infected cells (GFP positive cells, middle panels) and of the cell population gated as pDCs, single cells (CD123 positive cells, lower panels), are shown. Panels, as displayed from the left to the right, Bright field; GFP field; APC field; GFP/APC field and Merge. B. Quantification of the effect of heparin treatment (as in Fig 4B) on IFN-I production in SNs of pDCs co-cultured with HTLV-1-infected cells or HTLV-1-purified biofilm-like structure normalized to the amount of p19 measured in each biofilm-like structures preparation. The results are expressed as fold-increase relative to the untreated controls (mean ± SD; 10 and 3 independent experiments for HTLV-1 infected cells and biofilm-like structure, respectively). Asterisks indicate statistically significant differences calculated using ANOVA followed by Sidak’s multiple comparison test: *** p<0.001. (TIF) [file ppat.1007589.s004.tif]

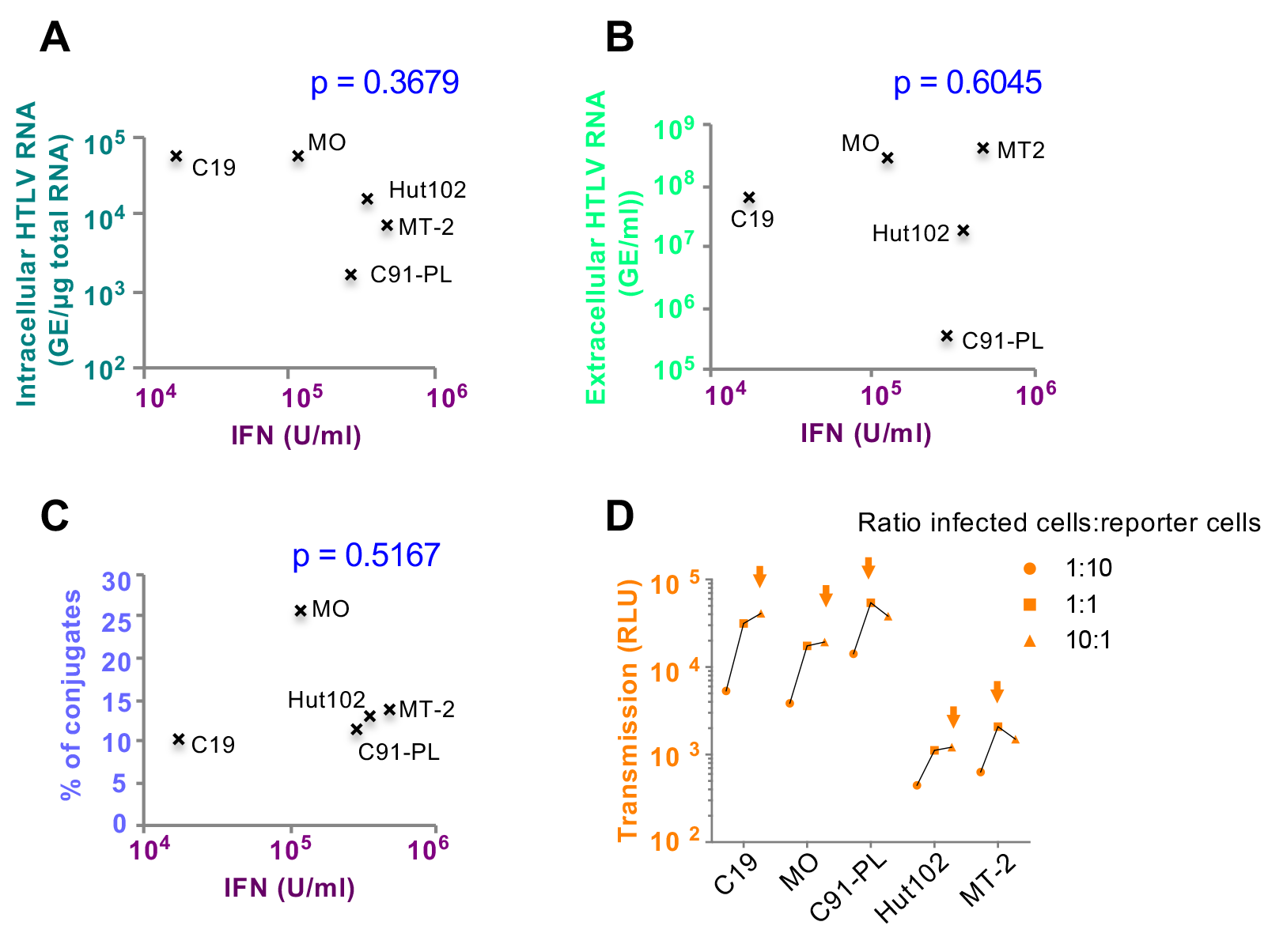

Supplement: S5 Fig — A-C. IFN-I amounts (U/ml) induced by HTLV- infected cells plotted against the corresponding intracellular RNA levels (A), extracellular RNA levels (B) or the percentage of cell-conjugates (C). Compute correlation p values are indicated. D. Infectivity levels determined after co-culture of Jurkat-LTR-Luc reporter cells (104 or 105) with HTLV-1 or HTLV-2 infected cells (104 or 105). The infected cells/reporter cell ratio (1:10 represents 104 infected cells for 105 reporter cells, 1:1 represents 105 infected cells for 105 reporter cells, 10:1 represents 105 infected cells for 104 reporter cells) is indicated on the right of the graph. RLU, relative light unit. Arrows indicate the maximum level of RLU relative to viral transmission for each cell line setting. (mean of 3 independent experiments). (TIF) [file ppat.1007589.s005.tif]

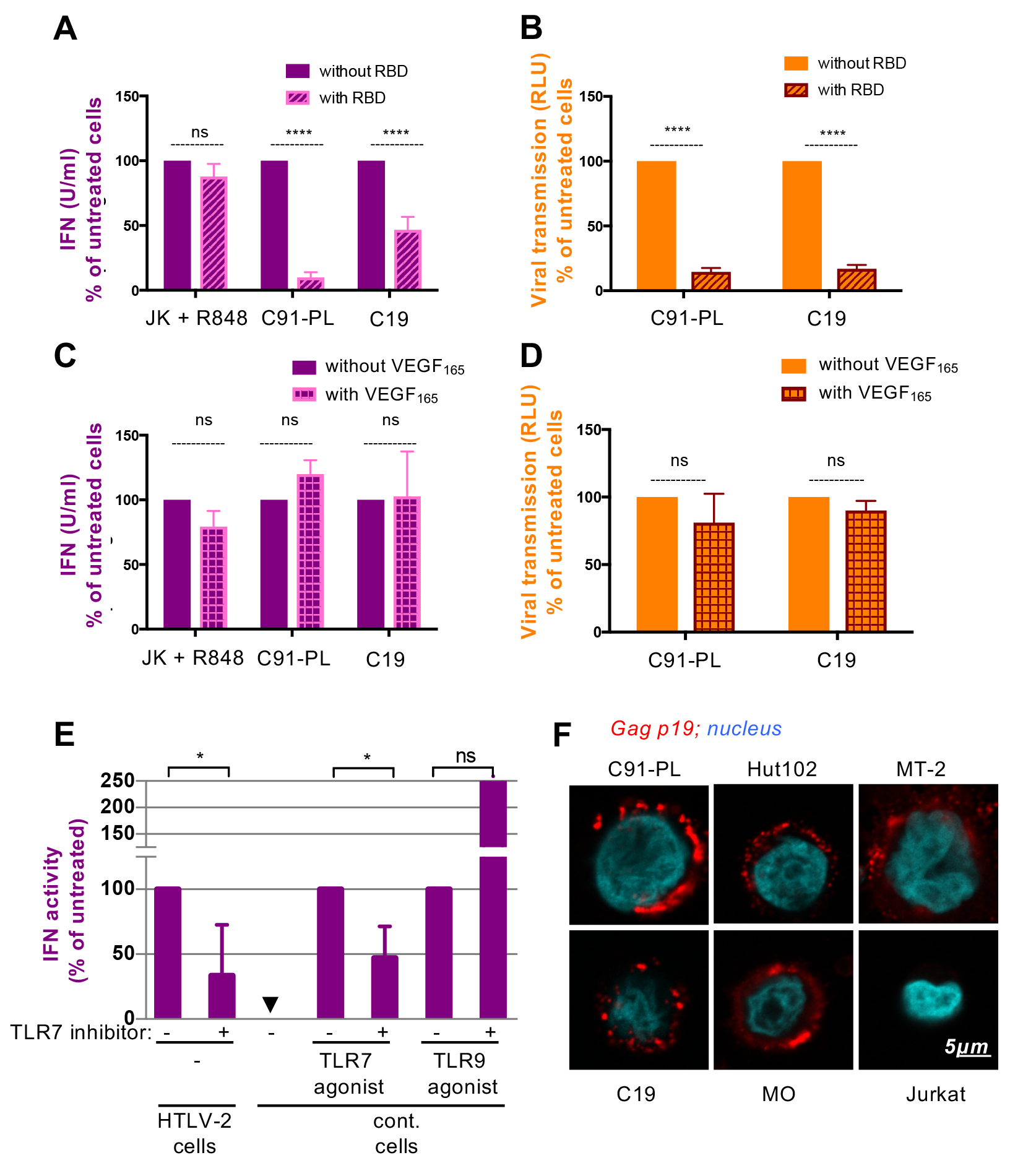

Supplement: S6 Fig — A and C. Impact of Glut-1 binding competitor (RBD, 5μL/105 cells, A) or NRP-1/BDCA-4 binding competitor (VEGF165, 100 ng/mL, C) on IFN-I activity in SNs of pDCs co-cultured with HTLV-1-infected cells (C91-PL) or HTLV-2 infected cells (C19). B and D Corresponding infectivity levels, determined as in Fig 2D. The results are expressed as percentages relative to untreated co-cultures (mean ± SD; 3–5 independent experiments). Asterisks indicate statistically significant differences calculated using ANOVA followed by Sidak’s multiple comparison test: **** p<0.0001; ns = non significant. E. Quantification of IFN-I activity in pDCs SNs. Cells were pre-incubated, or not, with TLR7 inhibitor (IRS661, 0.35 μM), as indicated, then co-cultured with infected cells HTLV-2 cells (C19 cells) or with control cells and stimulated by agonist of TLR7 (R848, 50 ng/mL) or of TLR9 (ODN2216, 0.1 μM). Results are expressed as percentages relative to IFN-I activity determined in the absence of TLR7 inhibitor, set at 100 (means ± SD; n = 3). F. Representative images (of 3 independent experiments) obtained by confocal microscopy of HTLV-1 (C91-PL; MT-2 or Hut102) or HTLV-2 (C19 or MO) -infected cells immunostained against p19gag (red) and counterstained with DAPI for nuclei (blue). Scale bar = 5μm. (TIF) [file ppat.1007589.s006.tif]

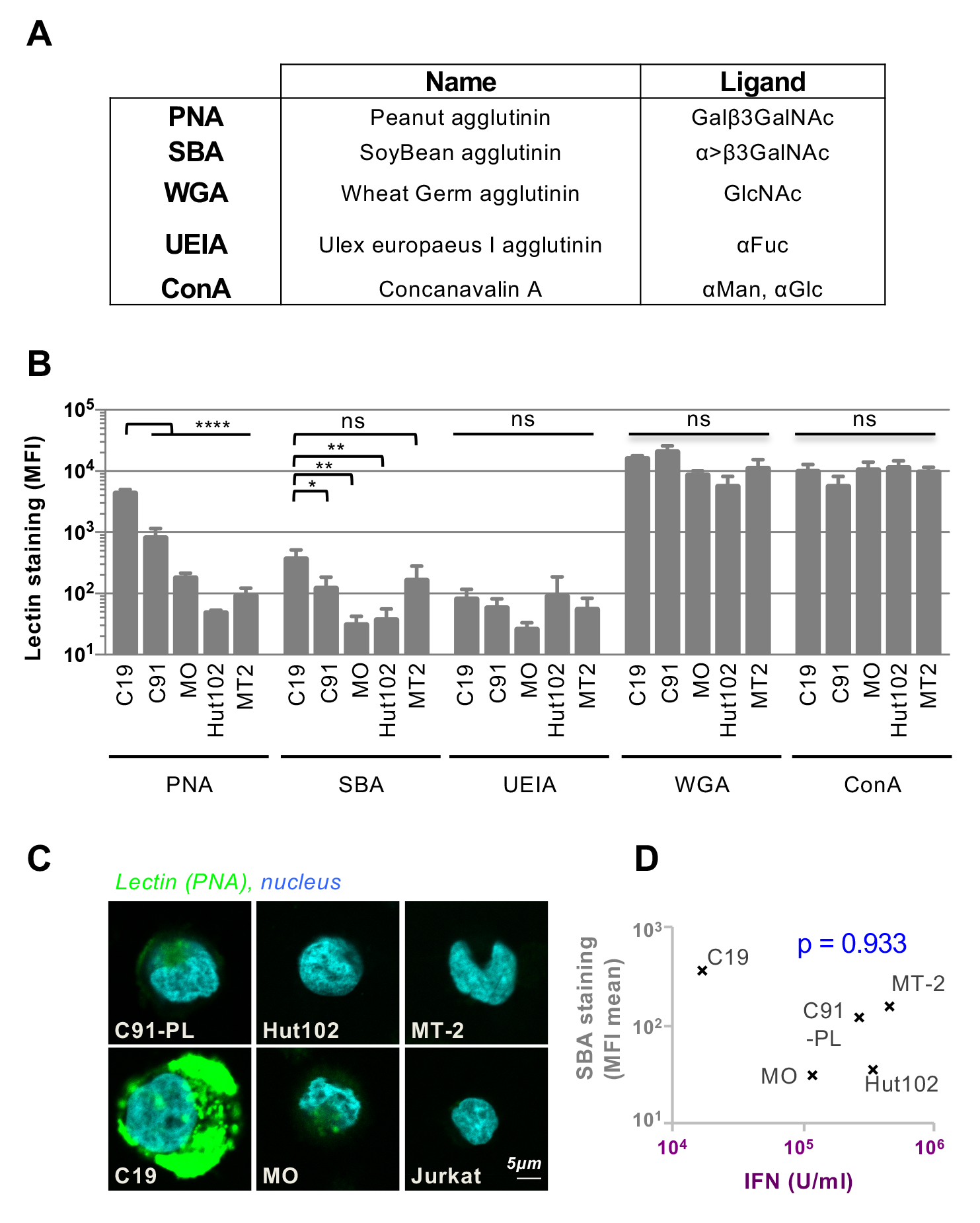

Supplement: S7 Fig — A. Name and binding specificity of the different lectins. B. Surface binding quantification of the various lectins on the HTLV-1/2 infected cells determined by FACS using FITC-coupled lectins. (Mean ± SD of 3 independent experiments). Asterisks indicate statistically significant differences calculated using ANOVA followed by Sidak’s multiple comparison test: * p<0.05; ** p<0.01; **** p<0.0001; ns = non significant C. Representative images obtained by confocal microscopy of HTLV-1- (C91-PL; MT-2 or Hut102) or HTLV-2- (C19 or MO) infected cells immunostained with FITC-coupled PNA (green) and DAPI for nuclei (blue). Scale bar = 5μm. D. Correlation curve of SBA expression (MFI) and pDC IFN-I production induced by coculture with HTLV-1 and HTLV-2 infected cells. Compute correlation p value is indicated. (TIF) [file ppat.1007589.s007.tif]

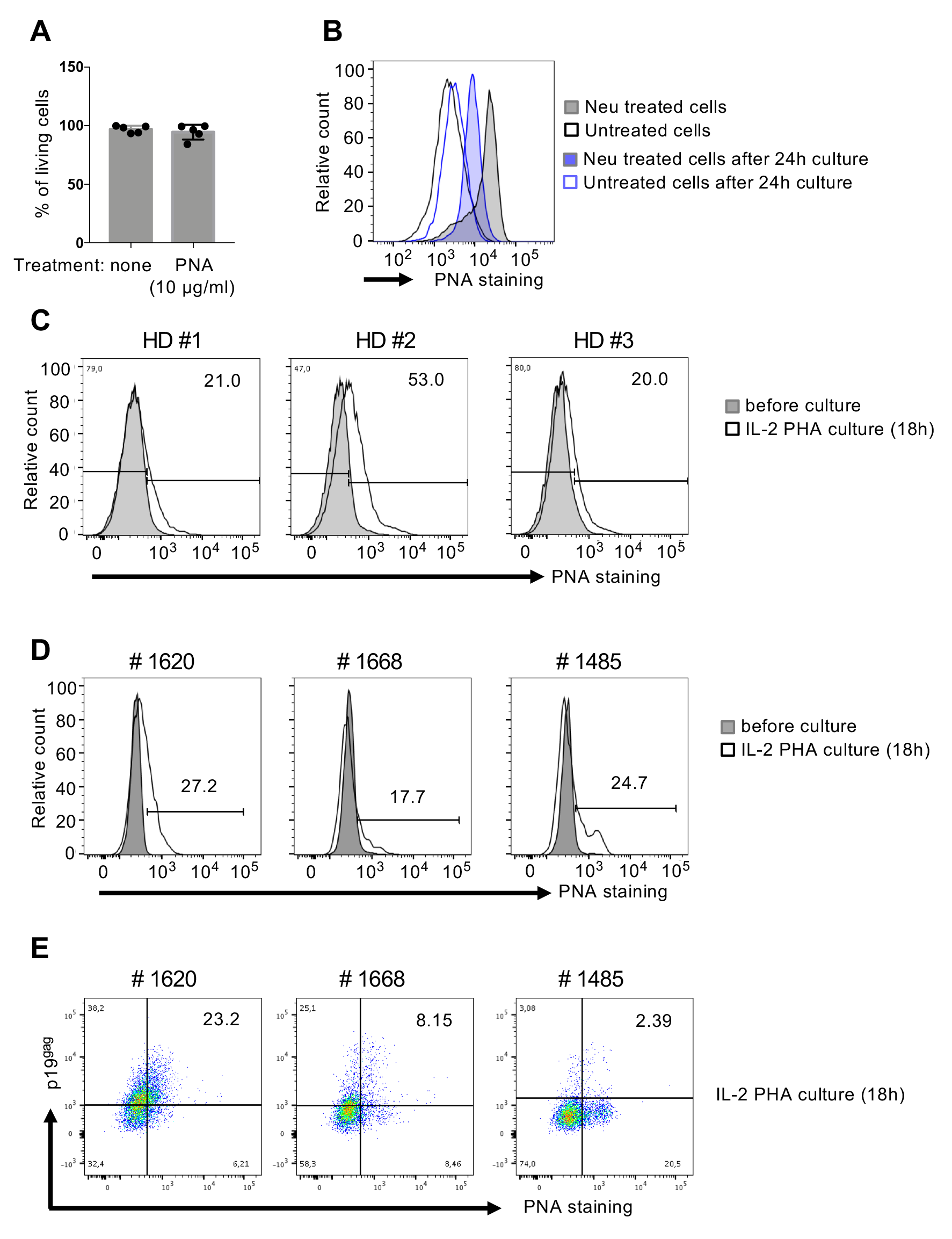

Supplement: S8 Fig — A. The percentage of living cells (i.e., aqua negative as measured by FACS using live-dead Aqua reagents from Thermofisher) was determined on C19 cells treated or not with PNA (10μg/ml) for 30 minutes (means ± SD; n = 5). B. C91-PL cells were treated (filled histograms) or not (unfilled histograms) with Neuraminidase (Neu, 0.1 U/ml) for 1h and were either immediately stained with PNA (grey histograms) or stained after 24h in vitro culture (blue histograms). Representative of 3 independent experiments. C. PBMCs from 3 independent healthy donors were stained with PNA before (grey histograms) or after 18h in vitro culture in presence of IL2 and PHA (white histograms). The percentage of PNA positive PBMCs is indicated on the right of each histograms. Representative of 3 independent experiments. D. PBMCs from 3 independent HAM/TSP patients were stained with PNA before (grey histograms) or after 18h in vitro culture in presence of IL2 and PHA (white histograms). The percentage of PNA stained PBMCs is indicated on each histogram. Representative of 3 independent experiments. E. PBMCs from 3 independent HAM/TSP patients were cultured for 18h in vitro in presence of IL2 and PHA. Viral expression and β-galactoside residues levels by co-staining with p19gag and PNA were determined by flow cytometry. The percentages of p19gag and PNA double-positive PBMCs are indicated on each plot. Representative of 3 independent experiments. (TIF) [file ppat.1007589.s008.tif]
